# Optimized breeding strategies to harness genetic resources with different performance levels

**DOI:** 10.1186/s12864-020-6756-0

**Published:** 2020-05-11

**Authors:** Antoine Allier, Simon Teyssèdre, Christina Lehermeier, Laurence Moreau, Alain Charcosset

**Affiliations:** 1 GQE - Le Moulon, INRAE, University Paris-Sud, CNRS, AgroParisTech, Université Paris-Saclay, 91190 Gif-sur-Yvette, France; 2 RAGT2n, Statistical Genetics Unit, 12510 Druelle, France

**Keywords:** Genetic resources, Genetic diversity, Genetic base broadening, Pre-breeding, Genomic prediction, Optimal cross selection

## Abstract

**Background:**

The narrow genetic base of elite germplasm compromises long-term genetic gain and increases the vulnerability to biotic and abiotic stresses in unpredictable environmental conditions. Therefore, an efficient strategy is required to broaden the genetic base of commercial breeding programs while not compromising short-term variety release. Optimal cross selection aims at identifying the optimal set of crosses that balances the expected genetic value and diversity. We propose to consider genomic selection and optimal cross selection to recurrently improve genetic resources (i.e. pre-breeding), to bridge the improved genetic resources with elites (i.e. bridging), and to manage introductions into the elite breeding population. Optimal cross selection is particularly adapted to jointly identify bridging, introduction and elite crosses to ensure an overall consistency of the genetic base broadening strategy.

**Results:**

We compared simulated breeding programs introducing donors with different performance levels, directly or indirectly after bridging. We also evaluated the effect of the training set composition on the success of introductions. We observed that with recurrent introductions of improved donors, it is possible to maintain the genetic diversity and increase mid- and long-term performances with only limited penalty at short-term. Considering a bridging step yielded significantly higher mid- and long-term genetic gain when introducing low performing donors. The results also suggested to consider marker effects estimated with a broad training population including donor by elite and elite by elite progeny to identify bridging, introduction and elite crosses.

**Conclusion:**

Results of this study provide guidelines on how to harness polygenic variation present in genetic resources to broaden elite germplasm.

## Background

Modern breeding has been successful in exploiting crop diversity for genetic improvement. However, current yield increases may not be sufficient in view of rapid human population growth [25]. Moreover, modern intensive breeding practices have exploited a very limited fraction of the available crop diversity [15, 50]. The narrow genetic base of elite germplasm compromises long-term genetic gain and increases the genetic vulnerability to unpredictable environmental conditions [39]. Efficient genetic diversity management is therefore required in breeding programs. This involves the efficient incorporation of new genetic variation and its conversion into short- and long-term genetic gain.

**Fig. 1:**
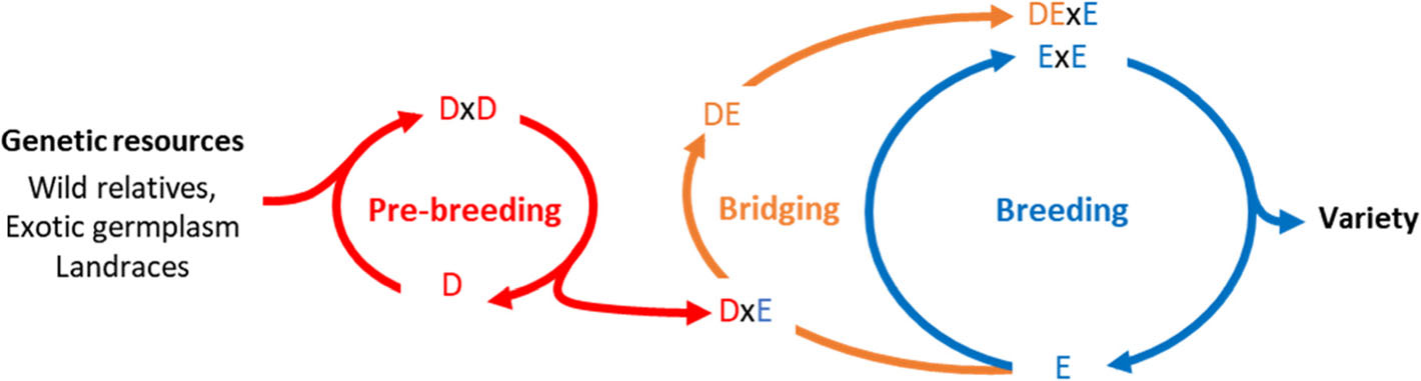
Diagram illustrating the respective positioning of pre-breeding, bridging and breeding from genetic resources to variety release

Among the possible sources of diversity, wild relatives, exotic germplasm accessions and landraces that predate modern breeding exhibit substantial genetic diversity. These ex-situ genetic resources are conserved worldwide in international gene banks and national collections. They provide a promising basis to improve crop productivity, crop resilience to biotic and abiotic stresses and crop nutritional quality [55, 72]. In case of traits determined by few genes of large effect, the favorable alleles can be identified and introgressed into elite germplasm following established marker-assisted backcross procedures (e.g. [13, 29, 58]). Such introgressions have been successful for mono- and oligogenic traits (e.g. earliness loci in maize, [60, 62] and SUB1 gene in rice, [8]). Introgressions also proved to be successful for more polygenic traits where few major causal regions have been identified. For instance, Ribaut and Ragot [51] successfully introgressed five regions associated with maize flowering time and yield components under drought conditions. For complex traits controlled by numerous genes with small effect, e.g. grain yield in optimal conditions, the identification and introgression of favorable alleles into elite germplasm were mostly unsuccessful [12]. This requires to go beyond the introgression of few identified favorable alleles toward the polygenic enrichment of elite germplasm [59, 61]. Although plant breeders recognize the importance of genetic resources for elite genetic base broadening, only little use has been made of it [24, 72]. The main reason is that breeding progress continues [20, 66] and that breeders are reluctant to compromise elite germplasm with unadapted and unimproved genetic resources [33]. Despite genetic resources carry novel favorable alleles that may counter balance their low genetic value by an increased genetic variance when crossed to elites [4, 37], their progeny performance is mostly insufficient for breeders. Thus, breeding strategies are needed to bridge the performance gap between genetic resources and elites and to transfer beneficial genetic variations into elite germplasm while not compromising the performance of released varieties [26, 61]. Pre-breeding can be defined as the recurrent improvement of diversity sources to release donors that can be further introduced into the elite breeding population (Fig. 1). According to Simmonds [61], pre-breeding should start from a broad germplasm and should be carried out on several generations with low selection intensity to favor extensive recombination events and minimal inbreeding. The donors released from pre-breeding can be directly introduced into the elite breeding population. However, in cases where the performance gap between the donors released from pre-breeding and elites is too large, one may consider a buffer population between donors and elites before introduction in the elite breeding population, further referred to as bridging. The best progeny of bridging is then considered for introduction into the elite breeding population (Fig. 1).

Different sources of donors can be considered for genetic base broadening. This includes landraces historically cultivated before modern breeding. For instance in maize, open pollinated varieties (OPVs) are landrace populations of heterozygous individuals cultivated before the hybrid maize breeding revolution in the 1950’s [7, 68]. Inbred lines derived from OPVs present a large diversity and a potential interest for adaptation, but also a large performance gap with current varieties [10, 11, 40]. These landraces can be further improved through pre-breeding that can be shared between the industry and public institutes in collaborative projects. In maize, the Latin American Maize Project (LAMP, [45, 54, 55]) provided breeders with useful characterization and evaluation of United State of America (US) and Latin American tropical germplasm accessions. Later, the Germplasm Enhancement of Maize project (GEM, [46]) improved the accessions identified in LAMP with elite lines furnished by private partners [47]. Similarly, the Seeds of Discovery project (SeeD, [26]) aimed to harness favorable variations from landraces and to develop a bridging germplasm useful for genetic base broadening of commercial maize breeding programs. In this vein, Cramer and Kannenberg [17] proposed the Hierarchical Open-ended Population Enrichment (HOPE) breeding system to release enriched maize inbreds for the industry. In its last version, the HOPE system is a breeding program with three hierarchical open ended gene pools permitting the transfer of favorable alleles from diversity sources to the elite pools [34, 48]. Finally, breeders can consider the varieties released by breeding programs selecting on a different germplasm and in different environments as donors. In species where hybrid varieties are cultivated, the ability to use one variety’s inbred parent as a donor depends on the germplasm proprietary protection relative to species and countries (e.g. the possibility of using reverse breeding, [63]). In the US, maize inbred parents of hybrid varieties become publically available after 20 years of plant variety protection act, these are referred to as ex-PVPA [44]. In inbred species such as wheat, using current varieties for breeding is straightforward if cultivated under the union for the protection of new varieties of plants convention (UPOV, [19]). These donors are likely the most performing but also the less original that can be considered.

With the availability of cheap high density genotyping, Whittaker et al. [73] and Meuwissen et al. [42] have proposed to use genomewide prediction to fasten breeding progress by shortening generation intervals. A large number of genomewide markers is employed, and their effects are estimated on a training set (TS) of phenotyped and genotyped individuals. The genomic estimated breeding values (GEBVs) are further predicted considering the estimated marker effects and individuals’ molecular marker information. Recurrent selection based on genomewide prediction, further referred to as genomic selection (GS), has been increasingly implemented in crop breeding programs [31, 70]. GS efficiency depends on the relationship between individuals in the TS and the target population of individuals to predict [28, 49]. As a consequence, in commercial breeding programs, GS has been mostly implemented considering a narrow elite TS that optimizes the prediction accuracy on elite material. However, such a narrow TS limits the prediction accuracy of individuals carrying rare alleles, which is the case for the progeny of elite by donor crosses. Therefore, it is important to define the TS composition that maximizes the prediction accuracy in both elite and introduction families.

In the context of genetic base broadening, GS is also interesting to fasten and reduce the costs for the evaluation and identification of genetic resources in gene banks [18, 77]. Furthermore, GS can fasten pre-breeding programs to reduce the performance gap between diversity sources and elite populations [26]. Instead of truncated selection (i.e. select and mate individuals with the largest estimated breeding values), Cowling et al. [16] proposed to use the optimal contribution selection to improve diversity sources while maintaining a certain level of diversity in the pre-breeding population. Optimal contribution selection [41, 74, 75] aims at identifying the optimal parental contributions to the next generation in order to maximize the expected genetic value in the progeny under a certain constraint on diversity. Therefore, the optimal contribution selection is particularly adapted to pre-breeding and genetic diversity management. Cowling et al. [16] considered the pedigree relationship information but genomic relationship information can further improve the optimal cross selection [14]. Considering optimal contribution selection on empirical cattle data, Eynard et al. [21] observed that allowing for the introductions of old individuals in the breeding population increased long-term response to selection. The optimal cross selection (OCS) is the extension of optimal contribution selection to deliver a crossing plan [1, 2, 27, 35, 36].

In this study, we propose to take advantage of OCS for selection of bridging, introduction and elite crosses (Fig. 1). Allier et al. [5] proposed to account for within family variance and selection in a new version of OCS referred to as Usefulness Criterion Parental Contribution based OCS (UCPC based OCS). UCPC based OCS differs from standard OCS in that it uses within-family variance to predict the expected mean performance and the expected genetic diversity in the selected fraction of the progeny while standard OCS predicts the expected mean performance and genetic diversity in the unselected progeny. Allier et al. [5] observed both higher short- and long-term genetic gain compared to OCS in a simulated closed commercial breeding program. We extend here the use of UCPC based OCS to pre-breeding, following Cowling et al. [16], and to an open commercial breeding program with recurrent introductions of diversity sources, extending the work of Eynard et al. [21]. Using OCS, the donor by elite crosses are selected complementarily to the elite by elite crosses in order to ensure an overall consistency of the genetic base broadening strategy. In this context, we aimed at evaluating the efficiency of genetic base broadening depending on the type of donors considered and the genetic base broadening scheme (Fig. 1). We considered either donors corresponding to the generation of the founders of breeding pools or improved varieties released 20 years ago and 5 years ago. Our objectives were to evaluate (i) the advantage of recurrent introductions of diversity in the breeding population compared to a benchmark scenario with no introduction, (ii) the interest to conduct or not bridging and (iii) the impact of the training set composition on within family genomewide prediction accuracies.

## Results

### Advantages of pre-breeding and bridging

The advantage of recurrent introductions in the commercial breeding program after or without bridging depended on the type of donor considered. Donors issued from a panel assembling founders of the breeding pool, referred to as panel donors, showed a large performance gap with the elites they were crossed to. This performance gap increased with advanced breeding generations (the true breeding value difference with elites increased from − 15 to − 104 trait units on average over the 60 years period). Improved donors showed a lower performance gap with elites. Twenty-year old donors showed an intermediate performance gap with elites (− 22 trait units on average over the 60 years period) and five-year old donors showed a reduced performance gap with elites (− 8 trait units on average over the 60 years period).

**Fig. 2:**
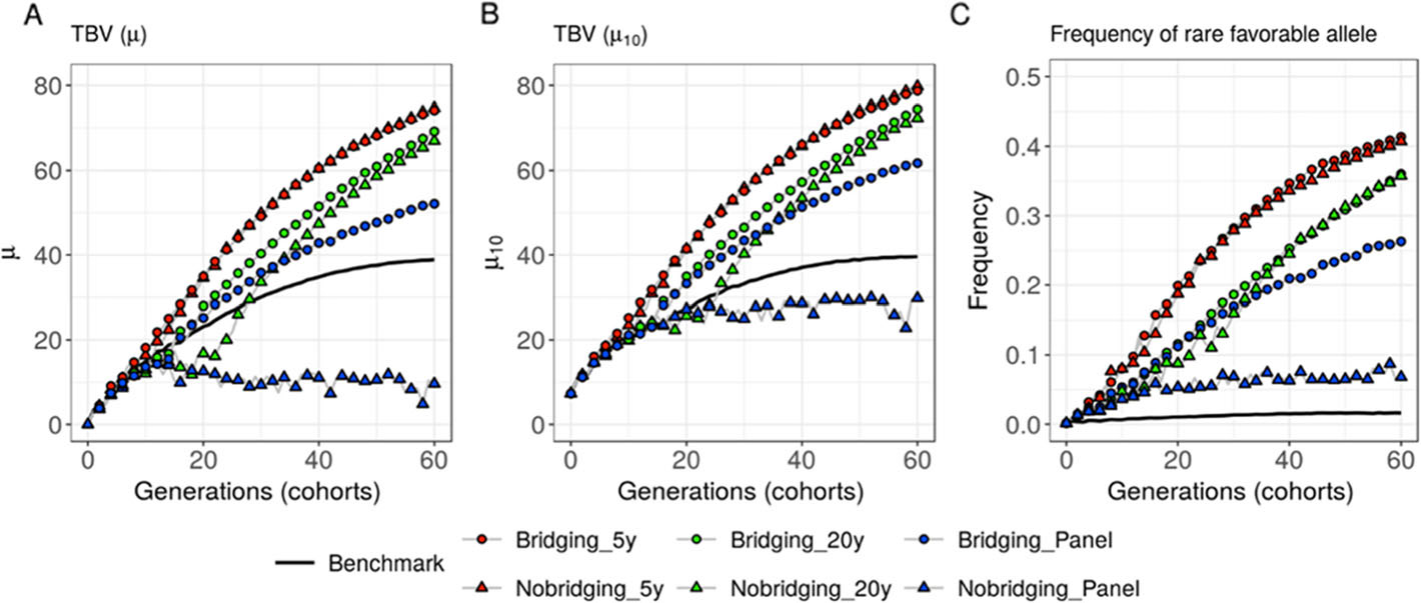
Evolution of the breeding population over generations. Scenarios considering presence or absence of bridging before introduction with different type of donors (panel, 20-year old and 5-year old donors). **a** Mean breeding population performance (*μ*), **b** mean performance of the 10 best progeny (*μ*_10_) and **c** frequency of the favorable alleles that were rare at the end of burn-in (i.e. *p*(0) ≤ 0.05 corresponding on average to 269.9 +/− 23.6 QTLs)

Direct introductions of panel donors without bridging (*Nobridging_Panel*) penalized the breeding population mean performance (*μ*) at short-term (at 5 years, *μ* = 8.168 +/− 0.282 compared to 9.239 +/− 0.237 without introductions, Fig. 2a, Table S1) and long-term (at 60 years, *μ* = 9.651 +/− 0.958 compared to 38.837 +/− 1.563 without introductions, Fig. 2a, Table S1). When considering the mean performance of the 10 best progeny (*μ*_10_), the short-term penalty was no more significant (at 5 years, *μ*_10_ = 15.802 +/− 0.341 compared to 15.746 +/− 0.391 without introductions, Fig. 2b, Table S2) but the long-term penalty was still significant (at 60 years, *μ*_10_ = 29.767 +/− 1.108 compared to 39.567 +/− 1.571 without introductions, Fig. 2b, Table S2). The introduction of panel donors after bridging (*Bridging_Panel*) did not significantly penalize the short-term mean performance of the breeding population (at 5 years, *μ* = 8.688 +/− 0.329 compared to 9.239 +/− 0.237 without introductions, Fig. 2a, Table S1) and yielded significantly higher long-term performance (at 60 years, *μ* = 52.110 +/− 0.886 compared to 38.837 +/− 1.563 without introductions, Fig. 2a, Table S1). When considering *μ*_10_, the short-term penalty was reduced (at 5 years, *μ*_10_ = 15.605 +/− 0.477 compared to 15.746 +/− 0.391 without introductions, Fig. 2b, Table S2) and the long-term gain increased (at 60 years, *μ*_10_ = 61.763 +/− 1.298 compared to 39.567 +/− 1.571 without introductions, Fig. 2b, Table S2).

Direct introductions of 20-year old donors without bridging (*Nobridging_20y*) yielded a penalty in the mid-term compared to not introducing donors (at 20 years, *μ* = 16.818 +/− 2.397 compared to 23.182 +/− 1.446 without introductions, Fig. 2a, Table S1). When considering *μ*_10_, the mid-term penalty due to introductions was limited (Fig. 2b, Table S2). After 30 years, this introduction scenario significantly outperformed the benchmark (*μ* = 33.546 +/− 1.519 compared to 30.006 +/− 1.319 without introductions, Fig. 2a, Table S1) and this advantage increased until the end of the 60 years evaluated period (*μ* = 66.944 +/− 0.849 compared to 38.837 +/− 1.563 without introductions, Fig. 2a, Table S1). The introduction of 20-year old donors after bridging (*Bridging_20y*) penalized only the short-term performance (at 5 years, *μ* = 8.687 +/− 0.293 compared to 9.239 +/− 0.237 without introductions, Fig. 2a, Table S1) and yielded significantly higher performance than the benchmark after 20 years (*μ* = 27.987 +/− 0.840 compared to 23.182 +/− 1.446 without introductions, Fig. 2a, Table S1). Introductions after bridging significantly outperformed the direct introductions until the end of the 60 years evaluated period (*μ* = 69.154 +/− 0.868 with bridging compared to 66.944 +/− 0.849 without bridging and *μ*_10_ = 74.413 +/− 0.932 with bridging compared to 72.258 +/− 0.978 without bridging, Fig. 2a-b, Table S1-S2).

Introducing 5-year old donors after or without bridging yielded significantly higher mid- and long-term performances than all other tested scenarios, without any significant long-term advantage of introductions after bridging compared to direct introductions (at 60 years, *μ* = 74.074 +/− 0.869 with bridging compared to 74.662 +/− 0.938 without bridging, Fig. 2, Table S1).

**Fig. 3:**
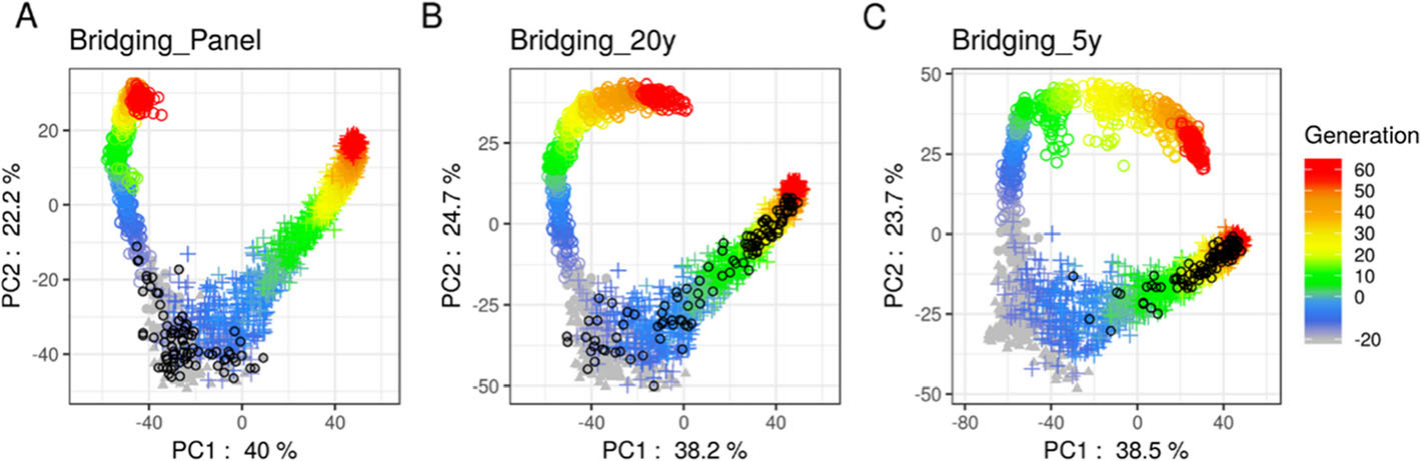
Principal component analysis of the modified Roger’s genetic distance matrix [76] of the 338 founders (gray: points for the 57 Iodent lines and triangles for the 281 remaining lines), the commercial 10 best performing E progeny per generation (colored circle sign) and the 20 donors per generation released by the external program (colored plus sign). Both commercial and external lines are colored regarding their generation (note that negative generations correspond to burn-in). Black circles represent the donors that have been introduced into the commercial breeding program. Only three scenarios with bridging are represented for the first simulation replicate, **a** when only donors from panel were accessible, **b** when 20-year old donors from the external breeding were accessible and **c** when 5-year old donors from the external breeding were accessible

We observed that the recurrent introductions of donors impacted the genetic diversity of the commercial germplasm. The faster the commercial program had access to recent germplasm of the external program, the more the varieties released by the commercial program were admixed with the external program elite germplasm (Fig. 3b and c). In the scenario where only panel donors were accessible for introductions, the internal program diversity did not converge toward the external program (Fig. 3a).

The evolution of the mean frequency of initially rare favorable alleles (i.e. favorable allele that had a frequency at the end of burn-in ≤0.05 in the elite breeding population) also highlighted differences between strategies. The older the donors, the lower the observed increase in frequency of initially rare favorable alleles (at 60 years for scenario with bridging, the mean frequency was 0.414 +/− 0.012 for 5-year old donors, 0.361 +/− 0.009 for 20-year old donors, 0.263 +/− 0.008 for panel donors and 0.016 +/− 0.006 without introductions, Fig. 2c, Table S3). For 20-year old donors, omitting the bridging before introduction delayed the increase in frequency of initially rare favorable alleles (e.g. at 20 years, the mean frequency was 0.088 +/− 0.014 without bridging compared to 0.116 +/− 0.011 with bridging, Fig. 2c, Table S3). For panel donors the absence of bridging significantly penalized the increase in frequency of initially rare favorable alleles (at 60 years, 0.068 +/− 0.007 without bridging compared to 0.263 +/− 0.008 with bridging, Fig. 2c, Table S3).

### Effect of a joint genomic selection model for bridging and breeding

**Fig. 4:**
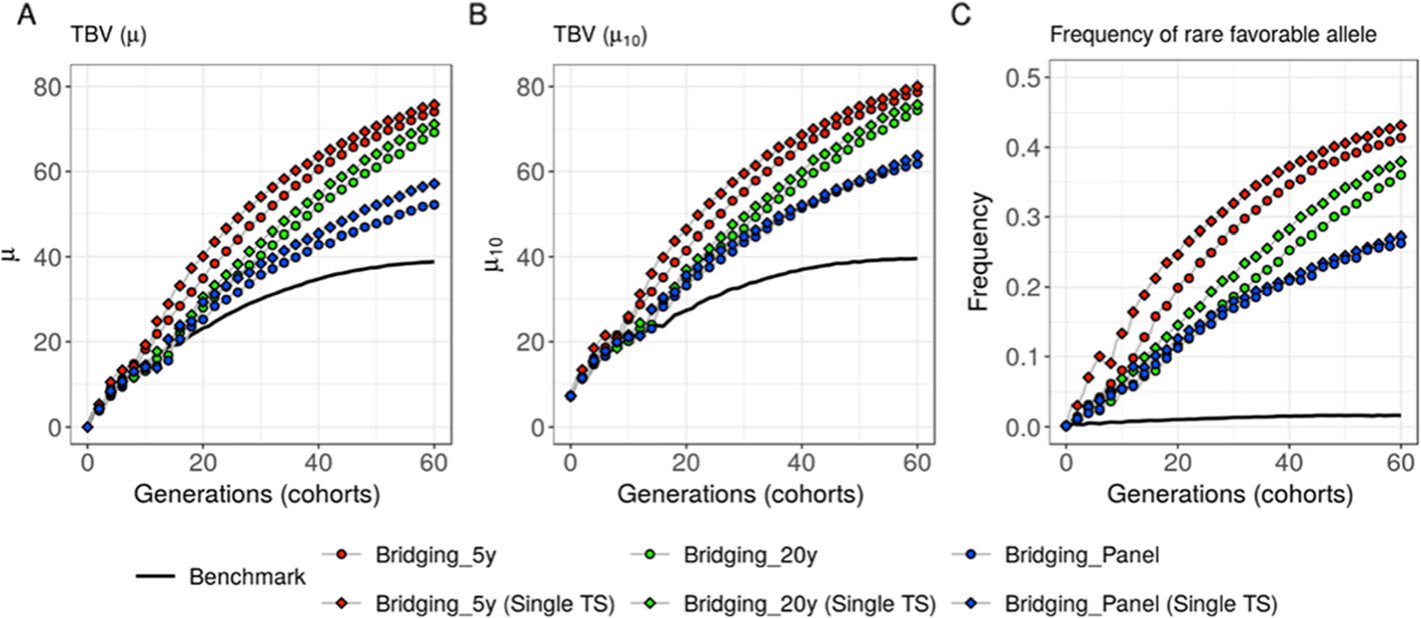
Evolution of the breeding population over generations. Scenarios considering bridging with different donors (panel, 20-year old and five-year old donors) and either a single broad TS (*Single TS*) or two distinct training sets for bridging and breeding (*default*). **a** Mean breeding population performance (*μ*), **b** mean performance of the 10 best progeny (*μ*_10_) and **c** frequency of the favorable alleles that were rare at the end of burn-in (i.e. *p*(0) ≤ 0.05 corresponding on average to 269.9 +/− 23.6 QTLs)

Scenarios with introductions after bridging that considered a single TS of 3600 E and 1200 DE progeny yielded higher mid- and long-term *μ* and *μ*_10_ than scenarios considering two distinct TS for bridging and breeding (Fig. 4a-b). After 20 years, single TS scenarios significantly outperformed scenarios with two distinct TS (*μ* = 40.111 +/− 1.149 compared to 34.900 +/− 0.905 for five-year old donors, *μ* = 30.497 +/− 1.135 compared to 27.987 +/− 0.840 for 20-year old donors and *μ* = 29.292 +/− 0.802 compared to 25.212 +/− 1.314 for panel donors, Fig. 4a, Table S1). After 60 years, the advantage of a single TS remained significant except for 5-year old donors (*μ* = 75.749 +/− 1.093 compared to 74.074 +/− 0.869 for 5-year old donors, *μ* = 71.130 +/− 1.028 compared to 69.154 +/− 0.868 for 20-year old donors and *μ* = 57.067 +/− 1.444 compared to 52.110 +/− 0.886 for panel donors, Fig. 4a, Table S1). When considering *μ*_10_, a single TS was still more performing but its interest was less significant (e.g. for panel donors after 60 years, *μ*_10_ = 63.699 +/− 1.698 compared to 61.763 +/− 1.298, Fig. 4b, Table S1-S2). A single TS also favored the increase in frequency of initially rare favorable alleles introduced by 5-year old donors and 20-year old donors (e.g. for 20-year old donors after 60 years, 0.380 +/− 0.010 compared to 0.361 +/− 0.009, Fig. 4c, Table S3).

The observed within family prediction accuracies varied depending on the TS considered. For 20-year old donors introduced after bridging, considering a single TS of 4800 DE + E did not significantly improve the prediction accuracy within ExE families compared to using the pure elite TS of 3600 E ($cor\left(u,\hat{u}\right)$ = 0.73 +/− 0.06 compared to $cor\left(u,\hat{u}\right)$ = 0.72 +/− 0.07, Table 1). However, it significantly improved the prediction accuracy within introduction DExE families compared to the pure elite TS of 3600 E ($cor\left(u,\hat{u}\right)$ = 0.77 +/− 0.07 compared to $cor\left(u,\hat{u}\right)$ = 0.61 +/− 0.11, Table 1). A single TS also slightly but not significantly improved the prediction accuracy within bridging DxE families compared to the pure bridging TS of 1200 DE ($cor\left(u,\hat{u}\right)$ = 0.78 +/− 0.05 compared to $cor\left(u,\hat{u}\right)$ = 0.73 +/− 0.06, Table 1). Similar observations were made on the other scenarios considering 5-year old and panel donors. Prediction accuracies were larger in introduction DExE and bridging DxE families with older donors, i.e. phenotypically distant to elites, due to larger within family variances (e.g. for DExE families 14.43 +/− 4.40 for panel donors, 6.92 +/− 2.10 for 20-year old donors and 5.00 +/− 1.41 for five-year old donors, Table 1).

**Table 1:** Within family prediction accuracies ($cor\left(u,\hat{u}\right)$) depending on the validation set (VS)

	Five-year old donor				Twenty-year old donor				Panel donor			
	Family variance	Prediction accuracy			Family variance	Prediction accuracy			Family variance	Prediction accuracy		
		TS = E (3,600)	TS = DE (1200)	TS = E + DE (4800)		TS = E (3,600)	TS = DE (1200)	TS = E + DE (4800)		TS = E (3,600)	TS = DE (1200)	TS = E + DE (4800)
VS = **ExE**	3.76 (1.17)	0.69 ^**a**^ (0.07)	0.48 (0.1)	0.72 ^**b**^ (0.06)	3.93 (1.06)	0.72 ^**a**^ (0.07)	0.47 (0.10)	0.73 ^**b**^ (0.06)	4.02 (1.16)	0.72 ^**a**^ (0.05)	0.44 (0.10)	0.73 ^**b**^ (0.05)
VS = **DExE**	5.00 (1.41)	0.60 ^**a**^ (0.1)	0.59 (0.1)	0.73 ^**b**^ (0.07)	6.92 (2.10)	0.61 ^**a**^ (0.11)	0.65 (0.10)	0.77 ^**b**^ (0.07)	14.43 (4.40)	0.65 ^**a**^ (0.12)	0.78 (0.07)	0.86 ^**b**^ (0.05)
VS = **DxE**	9.69 (2.01)	0.61 (0.08)	0.66 ^**a**^ (0.08)	0.73 ^**b**^ (0.07)	18.31 (3.78)	0.65 (0.08)	0.73 ^**a**^ (0.06)	0.78 ^**b**^ (0.05)	64.15 (12.89)	0.74 (0.07)	0.82 ^**a**^ (0.04)	0.86 ^**b**^ (0.03)

Elite (ExE), introduction (DExE) and bridging (DxE) and the training set (TS) considered: pure elite (E), pure bridging (DE) and merged (E + DE). Results are given for scenarios with different donors, from the panel, 20-year old and 5-year old donors, considering a single TS and prediction accuracies are averaged over the 10 replicates and all 60 generations. In brackets are given the standard errors averaged over 60 generations.

^a^ Prediction accuracies that would have been realized if the breeding (E) or bridging (DE) set had been each predicted only by the corresponding training set (to be compared with ^b^)

^b^ Realized prediction accuracies when considering a single training set (to be compared with ^a^)

**Fig. 5:**
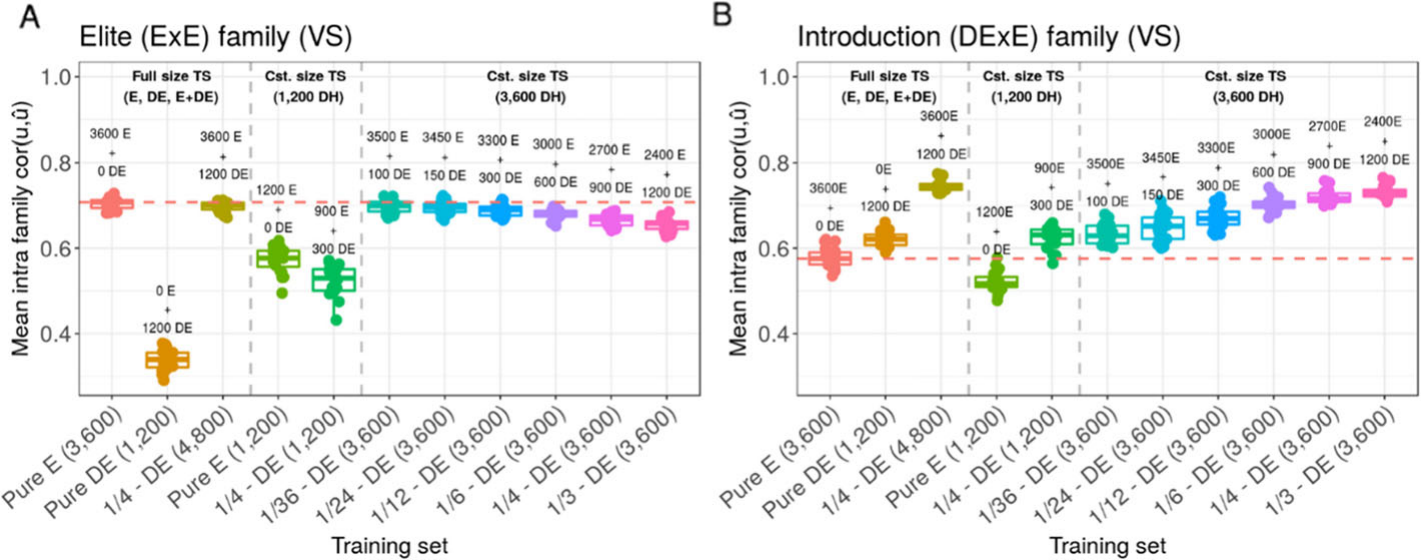
Effect of TS composition on intra family prediction accuracies ($cor\left(u,\hat{u}\right)$) considering genotypes simulated at generations 18, 19, 20 in the scenario *Bridging_20y*. **a** Mean prediction accuracy within 50 elite (ExE) families and **b** mean prediction accuracy within 50 introduction (DExE) families. Boxplots represent the results for 20 independent replicates. One can distinguish three training set types (left to right): Full training set considering all 3600 E progeny (Pure E), all 1200 DE progeny (Pure DE) and all 3600 E + 1200 DE progeny; Training sets at constant size of 1200 DH for comparison with Pure DE; Training sets at constant size of 3600 DH and variable proportion of DE progeny for comparison with Pure E. The red dotted line represents the median value for Pure E TS

**Fig. 6:**
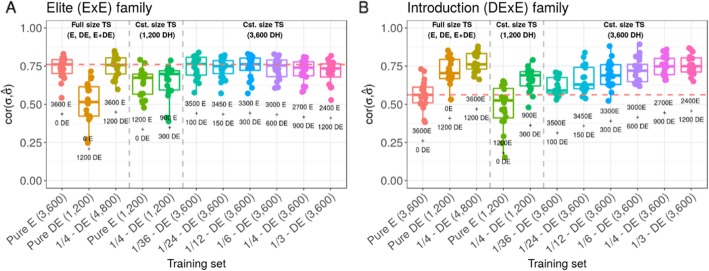
Effect of TS composition on family variance prediction accuracy ($cor\left(\sigma, \hat{\sigma}\right)$) considering genotypes simulated at generations 18, 19, 20 in the scenario *Bridging_20y*. **a** Mean prediction accuracy in 50 elite (ExE) families and **b** mean prediction accuracy in 50 introduction after bridging (DExE) families. Boxplots represent the results for 20 independent replicates. One can distinguish three training set types (left to right): Full training set considering all 3600 E progeny (Pure E), all 1200 DE progeny (Pure DE) and all 3600 E + 1200 DE progeny; Training sets at constant size of 1200 DH for comparison with Pure DE; Training sets at constant size of 3600 DH and variable proportion of DE progeny for comparison with Pure E. The red dotted line represents the median value for Pure E TS

At constant TS size of 3600 DH, the increase in proportion of DE progeny from 0 to 1/3 in the TS increased the prediction accuracy within introduction DExE families ($cor\left(u,\hat{u}\right)$ = 0.58 +/− 0.02 to 0.73 +/− 0.01, Fig. 5b) while it reduced the prediction accuracy within elite ExE families ($cor\left(u,\hat{u}\right)$ = 0.70 +/− 0.01 to 0.65 +/− 0.02, Fig. 5a). The TS with 3000 E and 600 DE appeared as a suitable compromise with within introduction DExE family $cor\left(u,\hat{u}\right)$ = 0.70 +/− 0.02 and elite ExE families $cor\left(u,\hat{u}\right)$ = 0.68 +/− 0.01. At constant TS size of 1200 DH, the TS with 900 E and 300 DE progeny performed similarly as the pure bridging TS for prediction within DExE families ($cor\left(u,\hat{u}\right)$ = 0.63 +/− 0.03 compared to 0.62 +/− 0.02, Fig. 5b) but significantly outperformed the pure bridging TS for prediction within elite ExE families ($cor\left(u,\hat{u}\right)$ = 0.52 +/− 0.04 compared to 0.34 +/− 0.02, Fig. 5a). The within family variance prediction accuracy showed similar tendencies (Fig. 6a-b). The increase in proportion of DE progeny from 0 to 1/3 in the TS increased the prediction accuracy within introduction DExE families ($cor\left(\sigma, \hat{\sigma}\right)$ = 0.56 +/− 0.09 to 0.76 +/− 0.07, Fig. 6b) while it slightly reduced the prediction accuracy within elite ExE families ($cor\left(\sigma, \hat{\sigma}\right)$ = 0.74 +/− 0.07 to 0.71 +/− 0.08, Fig. 6a).

## Discussion

Despite the recognition of the importance to broaden the elite genetic base in most crops, commercial breeders are reluctant to penalize the result of several generations of intensive selection by crossing elite material to unimproved diversity sources. Furthermore, among the large diversity available for genetic base broadening (e.g. landraces, public lines, varieties…), the identification of the useful genetic diversity to broaden the elite pool is difficult and might dishearten breeders. Consequently, there is a need for global breeding strategies to identify interesting sources of diversity that complement at best the elite germplasm, to improve diversity sources to bridge the performance gap with elites, and to efficiently introduce them into elite germplasm.

### Genetic base broadening with optimal cross selection accounting for within family variance

The identification of diversity sources for polygenic enrichment of the elite pool should account for the complementarity between diversity sources and elites as reviewed in Allier et al. [6]. Allier et al. [4] proposed the Usefulness Criterion Parental Contribution (UCPC) approach to predict the interest of crosses between diversity sources and elite recipients based on the expected performance and diversity in the most performing fraction of the progeny. The interest of UCPC relies on the fact that it accounts for within family variance and selection when identifying crosses. For instance, when crossing phenotypically distant parents, e.g. genetic resource and elite recipient, we expect a higher cross variance that should be accounted for to properly evaluate the usefulness of the cross [4, 37, 56]. Additionally, we expect the best performing fraction of the progeny to be genetically closer to the best parent. This deviation from the average parental value should be considered to evaluate properly the genetic diversity in the next generation [4, 5]. Accounting for parental complementarity at marker linked to QTLs also favors effective recombination in progeny and breaks negative gametic linkage disequilibrium between QTLs (i.e. repulsion), which unleashes additive genetic variance and increases long-term genetic gain [5]. Therefore, the OCS is particularly adapted to genetic diversity management in pre-breeding and breeding programs [1, 5, 16, 27]. Based on these studies, we evaluated a UCPC based OCS strategy to jointly select the donors and define the introduction and elite crosses to ensure an overall consistency of genetic base broadening accounting for the performance and diversity available in both bridging and breeding populations.

### Diversity sources and pre-breeding

Different sources of diversity can be considered by commercial breeders. The most original ones, but which show a large performance gap with elites, are landraces (e.g. DH libraries derived from landraces, [11, 40, 65]) and first varieties derived from landraces. Such a source of diversity was represented in our study by a fixed collection of panel donors. Since breeding industry is highly competitive, breeders are likely reluctant to introduce unselected sources of diversity directly into the breeding germplasm despite they might carry favorable adaptation alleles to face climatic changes [11, 30, 39]. Instead, commercial breeders will prefer to consider elite inbred lines from other than their own program [34].

In this study, the external breeding program was designed to release every generation several improved lines, later considered as donors for genetic base broadening of the commercial breeding program. The external program started from a broader genetic diversity than the commercial program (on average, He = 0.283 compared to He = 0.133 at the end of burn-in) and was designed to maintain higher genetic diversity during selection (on average, He = 0.101 compared to He = 0.014 after 60 years). This was done to mimic in a simple way the outcome of the activity of several companies conducting separate programs and therefore maintaining a global diversity. The external program can also be viewed as a pre-breeding program since it aimed at improving diversity sources to reduce the performance gap with elites while maintaining genomewide diversity (Fig. 1). The situation where the commercial breeding program can access donors released 20 years ago mimicked the situation of private lines with expired plant protection act in maize [44] or old public lines. The situation where the commercial breeding program can access donors released 5 years ago mimicked either donors released by pre-breeding programs (e.g. in maize the SeeD project, [26]) or donors released by programs working a different genetic basis and targeting different environments (e.g. commercial varieties in inbred species such as wheat that are accessible for breeding under the UPOV convention, [19]).

The selection intensity was lower in the external breeding than in the commercial breeding programs (10% vs 5% of progeny selected, respectively). This was done to compensate the increased response to selection due to the higher genetic diversity and ensure that the donors released by the external program underperform the commercial breeding elites. It should be noted that donors outperforming elites might be encountered in practice when considering elite germplasm as source of diversity, but this situation was not considered in this study. In such a situation the direct introduction of donors would be clearly preferable.

Our results highlighted a clear beneficial effect of introducing external diversity in the elite program. This benefit increased with increasing performance level of the introduced material from unimproved genetic resources collections (panel donors) to recently improved diversity sources (5-year old donors). This highlights that protection policies that permit a mildly delayed access to improved competitor varieties as diversity sources have a positive impact on long term genetic gain. This also shows that recurrent improvement of diverse and low performing genetic resources such as landraces, i.e. pre-breeding, may be beneficial before introduction in the elite germplasm. More importantly, we show that the approach for introduction should be tuned given the type of external diversity that can be accessed (see next section).

### Advantages of bridging relative to direct introductions in the elite pool

When considering recent donors (5-year old), scenarios with introductions after bridging or direct introductions performed similarly. Conversely, for panel and 20-year old donors, introductions after bridging yielded significantly higher mid- and long-term performance compared to direct introductions.

For panel donors showing a large performance gap with elites, the direct introductions were not converted into genetic gain. The high inter-family additive variance in this scenario (Figure S1 A) reflected the structuration of the breeding population into badly performing introduction families and performing elite families with only limited gene flow between them. Such behavior might be corrected by adding a constraint to force the recycling of introduction progeny in Eq. 1 (see Methods section) when donors are too badly performing, which requires further investigations. Waiting for these developments, bridging seems a suitable option to take advantage of donors that show a large performance gap with elites.

For donors with an intermediate gap level, both direct introductions and bridging brought a higher long-term genetic gain compared to the benchmark. This advantage was higher with bridging. More importantly, bridging reduced to a large extent the short term penalty in genetic gain which was observed for direct introductions. So here again bridging appears as a suitable option to maximize efficiency at different time horizons.

These results can be explained by the fact that, when donors (D) were less performing than elites, the fraction of progeny selected in donor by elite bridging families (DE progeny) carried on expectation less than half of donor’s genome [4]. Thus, progeny of introduction crosses after bridging (DExE) carried on expectation less than one fourth of the donor (D) genome. This selected D fraction carried an enrichment in favorable alleles but also still unfavorable alleles brought by linkage drag, which number depends on the donor considered. Introductions penalized slightly the mean breeding population performance in the first generations (Fig. 2a-b). Next generations of recombination and selection partially broke the linkage between favorable and unfavorable alleles in introduced regions, resulting in a higher genetic gain than in the benchmark (Fig. 2a-b) and an increase of the frequency of novel favorable alleles (Fig. 2c). The more performing the donor, the less unfavorable alleles linked to favorable alleles and the more rapidly novel favorable alleles were introduced and spread in the breeding population (Fig. 2c). In absence of bridging, the introduction progeny (DxE) carried on expectation one half of the donor genome. Consequently, the penalty due to introductions was more important and the conversion of additional diversity into genetic gain required more recombination events, i.e. recycling generations (Fig. 2a-b). In a practical breeding context, in absence of explicit bridging, the crosses DExE will be delayed in time compared to scenario with bridging. Incorporation of diversity contributed by donors requires, in this case, that DE progeny of previous generations are given the opportunity to contribute to next generations despite their lower performances.

### Practical implementation in breeding programs

We considered a commercial breeding program with a genetic diversity matching that of an experimental program reported by Allier et al. [3]. Breeding programs ongoing for different species and breeders may present a diversity superior or inferior to the one that was simulated, which would make the importance of introductions lower or stronger than in the simulated scenarios, respectively. UCPC based OCS for genetic base broadening requires to genotype the candidate parents, including breeding material and potential donors, a genetic map and reliable marker effect estimates. This information is available in breeding programs that have already implemented genomic selection. In this study, we assumed fully homozygous inbred lines but considering heterozygote parents in UCPC based OCS is straightforward following the extension of UCPC to four-way crosses [4]. So similar approach could be tested for perennial plants or animal breeding schemes.

In scenarios with bridging, we considered by default two distinct bridging and breeding GS models. The prediction of elite (ExE) and introduction (DExE) crosses usefulness and the prediction within crosses were based on a model trained on the breeding progeny of the three corresponding previous generations. Considering a unique genomic selection model trained on both bridging and breeding progeny increased the prediction accuracy within introduction families (DExE) (Table 1). This higher selection accuracy favored the spreading of the introduced favorable alleles in the breeding population and resulted in an increased mid- and long-term performance (Fig. 4). Furthermore, compared to use two distinct TS, a single TS led to introduce more bridging progeny (DE) for scenarios considering good performing donors (5-year old) and less for scenarios considering bad performing donors (20-year old) (Figure S2 A). Also, as we likely selected more accurately the introduction crosses (DExE) with a single TS, there was an increase in the proportion of those that contributed to the 10 best lines, especially for 20-year old and panel donors (Figure S2 B).

It is well known that the prediction accuracy is increased for larger TS [32]. At constant TS size, increasing the proportion of bridging progeny (DE) up to one third in the TS significantly increased the family variance prediction accuracy ($cor\left(\sigma, \hat{\sigma}\right)$) and within family prediction accuracy ($cor\left(u,\hat{u}\right)$) in introduction families (DExE). Conversely, these higher proportions of bridging progeny (DE) in the TS significantly decreased $cor\left(\sigma, \hat{\sigma}\right)$ and $cor\left(u,\hat{u}\right)$ in elite families (ExE). The optimal balance between introduction and elite family prediction accuracies is likely data dependent as observed when considering genotypes and phenotypes simulated in different generations (Figure S3). For instance, considering later generations, a large proportion of DE in the TS penalized less the within elite prediction accuracy (Figure S3 C). The reason being that later breeding generations get closer to the external program germplasm (Fig. 3). The optimal balance between bridging and breeding progeny in the training set might be defined using an optimization criterion such as the CDmean [52] extended to account for linkage disequilibrium as suggested by Mangin et al. [38].

We proposed to implement bridging at constant cost by splitting the breeding population into a small bridging population and a large breeding population. This involves practical changes in the breeding organization that remain to be studied. We considered equal family sizes and within family selection intensities for bridging and breeding families. However, in practice different within family selection intensities can be considered in UCPC based OCS (Additional file 2) and one may want to modulate the selection intensity among families, e.g. select less intensively in bridging and more intensively in breeding families. We could consider the selection intensities as fixed parameters regarding breeding objectives or as variable parameters to be optimized. The effect and the optimization of within family intensities in bridging and breeding requires further investigations. We considered a selection accuracy *h* = 1 for cross selection, for sake of facility. However, we observed that within family prediction accuracies were variable (Table 1, Fig. 5). Note that a priori within family accuracy can be accounted for in UCPC based OCS (Additional file 2). For instance it would give less importance to predicted variance for crosses with a priori low within family accuracy. The consequences on short- and long-term UCPC based OCS efficiency need to be investigated. In bridging, we gave more importance to performance than to diversity (*α* = 0.7) when selecting bridging crosses in order to reduce the performance gap between donors derived material and elites. When giving less weight to the performance than to the diversity, i.e. *α* = 0.3, we observed non-significant changes on the short- or long-term performance for scenarios with 5-year and 20-year old donors and a significant increase of long-term performance and novel favorable allele frequency for the scenario with panel donors (Figure S4 A-C). This suggested that for unimproved donors, selecting too strongly for performance in bridging favors the first elite recipient genome contribution and limits the introduction of novel favorable alleles. Further investigations are required to better define this parameter for practical implementation.

### Outlooks

We considered an inbred line breeding program corresponding to selecting lines on per se values for line variety development or on testcross values with fixed tester lines from the opposite heterotic pool for hybrid breeding. In this case, the use of testcross effects estimated on hybrids between candidate lines and tester lines is straightforward. The extension to hybrid reciprocal breeding is of interest for genetic broadening in several species such as maize and hybrid wheat [37]. In this context it is possible to account for the complementarity between heterotic groups in UCPC based OCS to complementarily enrich and improve both pools. This would require to include dominance effects in UCPC based OCS.

We assumed that diversity sources and elite germplasm were derived from the same panel of founders and shared similar QTL effects. In order to address the question of how to best introduce more exotic genetic material that might not be adapted to local conditions, it would be necessary to take into account potential differences in QTL effects and linkage between QTLs and markers.

We considered a single trait selected in both the external and the commercial breeding programs in the same population of environments for a total of 8 years. These assumptions should be relaxed in further simulations. Firstly, it is well recognized that genetic resources suffer agronomic flaws (e.g. lodging, [37, 67]) or miss adaptation (e.g. flowering time) that should be accounted for during pre-breeding and introduction in breeding. Also, in addition to new grain yield favorable alleles, diversity sources can provide elite germplasm with increased stress tolerance and improved nutritional and processing quality (e.g. in wheat, [57]). In species where major genes are routinely followed in breeding (e.g. baking quality in wheat, [9, 43]), they should also be considered during pre-breeding and introduction in elite germplasm. In such a multi-trait context, the multi-objective optimization framework proposed in Akdemir et al. [2] can be adapted to UCPC based OCS. This would require further investigations but we assume the observed tendencies between simulated scenarios should remain. Secondly, in practice several public pre-breeding programs or competitor programs can be considered as sources of candidate donors for genetic base broadening. These programs likely did not select for the same target environments and are themselves continuously enriched in new allelic variation. Thirdly, in a context of climate change and rapid evolving agricultural practices, breeding targets are expected to change (e.g. emerging biotic or abiotic stresses). Considering a more realistic context, where donors are released by different programs selecting in different environments and for different traits changing over time, likely makes the interest of maintaining genomewide genetic diversity through genetic base broadening even more important than highlighted in this study.

## Conclusions

This study highlights a clear beneficial effect of harnessing polygenic variation present in diversity sources to broaden the elite genetic pool, while still achieving significant genetic gain. This interest is all the more important as the level of introduced material is high, which highlights the importance of pre-breeding and the effect of plant protection policies. We show that the strategy for introduction should be tuned given the type of external diversity that can be accessed. This study provides a guideline for reaching an optimized genetic base broadening strategy.

## Methods

### Simulated breeding programs

#### Material and simulations

We considered 338 Dent maize genotypes from the Amaizing project [6, 53] as founders of genetic pools. This diversity was structured into three main groups: 82 Iowa Stiff Stalk Synthetics, 57 Iodents and 199 other dents. We sampled 1000 biallelic quantitative trait loci (QTLs) with a minimal distance between two consecutive QTLs of 0.2 cM among the 40,478 single nucleotide polymorphisms (SNPs) from the Illumina MaizeSNP50 BeadChip [22]. Each QTL was assigned an additive effect sampled from a Gaussian distribution with a mean of zero and a variance of 0.05 and the favorable allele was attributed at random to one of the two SNP alleles. We sampled 2000 SNPs as non-causal markers, further used as genotyping information. The consensus genetic positions of sampled QTLs and SNPs were considered according to Giraud et al. [23].

**Fig. 7:**
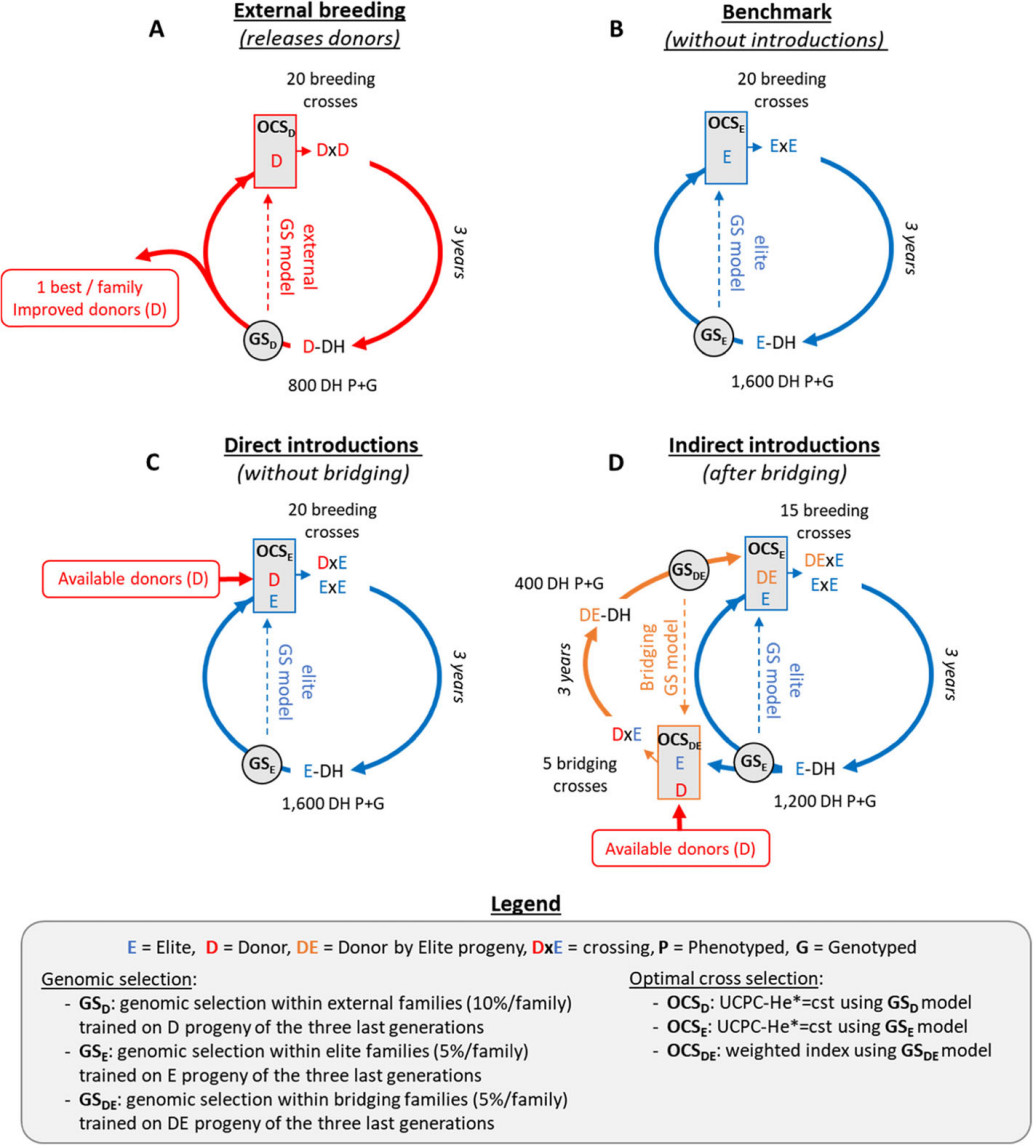
Diagram of simulated breeding programs. **a** External breeding program that generates potential donors, **b** commercial benchmark program without introductions, **c** commercial program with introductions without bridging or **d** commercial program with introductions after bridging

Simulation parameters were first applied to the 338 founders, to define a fixed collection of genetic resources that can be accessed to retrieve genetic diversity. This simulates the status of genetic resources collections which are not enriched by regular inputs from breeding programs. Then, we simulated two different breeding programs: an external breeding program (Fig. 7a) that released every year varieties that were later considered as potential donors for introduction in a commercial breeding program (Fig. 7c-d). Both external and commercial programs used doubled haploid (DH) technology to derive progeny. We assumed a period of 3 years to derive, genotype and phenotype DH progeny. Every year *T*, progeny of the three last generations *T*− 3, *T*− 4 and *T*− 5 were considered as potential parents of the next generation. It created overlapping and connected generations as it can be encountered in breeding. We first considered a burn-in period of 20 years with recurrent phenotypic selection from a population of founders. Burn-in created extensive linkage disequilibrium as often observed in elite breeding programs [69]. Every progeny was phenotyped and phenotypes were simulated considering the genotypes at QTLs, an error variance corresponding to a trait repeatability of 0.4 in the founder population, and no genotype by environment interactions (Additional file 1). Every individual was evaluated in four environments in one year. After 20 years of burn-in, we simulated different breeding programs using GS. Every year, progeny phenotypes and genotypes of the three last available generations were used to fit a G-BLUP model (Additional file 1). Progeny were selected based on GEBVs and marker effects were obtained by back-solving the G-BLUP model [71] and further used for optimal cross selection to generate the next generation (see Additional file 2).

#### External breeding program: improvement of diversity sources

The external breeding program (Fig. 7a) was simulated starting from a broad population of 40 founders sampled among the 338 maize genotypes. During the three first years, the founders were randomly crossed with replacement to generate each year 20 biparental families of 40 DH progeny to initiate the three overlapping generations. The genetic material in the external breeding is referred to as improved donors (D). During 17 years, we first selected among the three last generations the 10% D progeny per family (i.e. 4 DH lines/family × 20 families × 3 years) with the largest phenotypic mean. We further randomly mated with replacement the 50 DH with the largest phenotypic mean to generate 20 biparental families of 40 DH lines. After 20 years of burn-in, we considered GS trained on the D progeny of the three last generations (i.e. 2400 D progeny, Fig. 7a). Among these three last generations, we considered per family the 10% D progeny with the largest GEBVs as potential parents of the next generation, i.e. N_D_ = 4 DH lines/family × 20 families × 3 years = 240 potential parents. The 20 two-way crosses among the N_D_(N_D_-1)/2 = 28680 candidate crosses were selected using optimal cross selection (see optimal cross selection section).

#### Commercial breeding programs

The commercial breeding program (Fig. 7b-d) started from a population of 10 founders sampled among the 57 Iodent genotypes. During the first 3 years, the founders were randomly crossed with replacement to generate each year 10 biparental families of 80 DH progeny to initiate the three overlapping generations. The elite genetic material in the internal breeding is referred to as elite progeny (E). During 17 years, we considered as potential parents of the next generation the 50 E progeny with the largest phenotypic mean from the three last generations, i.e. without applying a preliminary within family selection. These were randomly mated to generate 20 biparental families of 80 DH lines. After 20 years of burn-in, we considered GS and differentiated three different scenarios: the benchmark that is the commercial breeding program without introductions (Fig. 7b), the commercial breeding program with direct introductions without bridging (Fig. 7c) or the commercial breeding program with introductions after bridging (Fig. 7d).

In absence of introductions (*benchmark*), the E progeny were selected based on the elite GS model trained on E progeny of the three last generations (i.e. 4800 E progeny, Fig. 7b). The 5% E progeny with the largest GEBVs within each family (i.e. 4 DH) in the three last breeding generations were considered as potential parents. The 20 two-way crosses among the 28680 candidate ExE elite crosses were defined using optimal cross selection (see next section).

For scenarios with introductions, we considered different sub-scenarios for the genetic base broadening scheme (i) including (*Bridging*) or not bridging (*Nobridging*) and (ii) different types of potential donors, to cover different possibilities in both hybrid and inbred species. We considered as potential donors either the 338 genotypes from the Amaizing project or the D progeny with the largest GEBVs per family released by the external breeding program (i.e. 1 DH/family/year, 20 potential donors released every year). The scenario using the 338 genotypes from the Amaizing panel for genetic base broadening was identified with the suffix *Panel*. For the donors released by the external breeding program, we considered two time constraints for the access to diversity. To mimic a situation close to that of the US maize ex-PVPA system [44], we considered donors released 20 to 24 years before the current year (i.e. 5 years × 20 DH = 100 potential D) in scenarios with the suffix *20y*. To simulate a faster access to external diversity, as it would be the case in line breeding under UPOV convention [19], we considered the donors released by the external breeding 5 to 9 years before the current year (i.e. 100 potential D) in scenarios with the suffi× *5y*.

For scenarios without bridging (Fig. 7c), the E candidate parents were selected every year among the 5% E progeny showing the largest GEBVs per family in the three last breeding generations resulting in N_E_ = 4 DH × 20 families × 3 years = 240 potential E parents. The E progeny were selected based on the elite GS model trained on E progeny of the three last generations (i.e. 4800 E progeny, Fig. 7c). The 20 breeding crosses among the 28680 candidate ExE elite crosses and the candidate DxE introduction crosses were selected using optimal cross selection and the elite GS model (see next section). Note that there was no constraint on the proportion of ExE elite or DxE introduction crosses.

For scenarios with bridging (Fig. 7d), the population was split into a bridging population of 5 families of 80 DH (i.e. 400 DE progeny) and a breeding population of 15 families of 80 DH (i.e. 1200 E progeny). Every year, the 15 breeding crosses were selected among all possible ExE elite and DExE introduction crosses. The E candidate parents for breeding were selected among the 5% E progeny per family showing the largest GEBVs from the three last breeding generations, resulting in N_E_ = 4 DH/family × 15 family × 3 year = 180 potential E parents. The E progeny were selected based on the elite GS model trained on all E progeny of the three last generations (i.e. 3600 E progeny, Fig. 7d). The DE candidate parents for introduction in the breeding population were similarly selected among the three last bridging generations, resulting in N_DE_ = 4 DH/family × 5 families × 3 years = 60 potential DE parents. The DE progeny were selected based on the bridging GS model trained on all DE progeny of the three last generations (i.e. 1200 DE progeny, Fig. 7d). Among the N_E_(N_E_ -1)/2 = 16110 ExE possible elite crosses and the N_DE_N_E_ = 10800 DExE possible introduction crosses, 15 breeding crosses were selected using optimal cross selection with the elite GS model (see next section). Note that there was no constraint on the proportion of ExE elite or DExE introduction crosses. The 5 DxE bridging crosses were selected with the bridging GS model among the possible crosses between the available D and the E candidate parents conditionally to the 15 selected breeding crosses (see next section).

### Optimal cross selection

The optimal cross selection selects the set of crosses (***nc***) that maximizes the expected genetic value in the progeny (*V*) under a constraint on the genomewide genetic diversity in the progeny (*D*) [1, 2, 27, 35, 36]. As proposed in Allier et al. [5], the effect of within family selection with intensity (*i*) and accuracy (*h*) on *V*^(*i*, *h*)^ and *D*^(*i*, *h*)^ can be accounted for in optimal cross selection by using UCPC based OCS (Additional file 2). Similarly as in Allier et al. [5], we considered *h* = 1 for sake of simplicity.

For breeding crosses, the optimal set of ∣***nc***∣ = 20 crosses (in scenarios without bridging, Fig. 7a-c) or ∣***nc***∣ = 15 crosses (in scenarios with bridging, Fig. 7d) was selected to solve the multi-objective optimization problem:

$$ \underset{\boldsymbol{nc}}{\max }{V}^{(i)}\left(\boldsymbol{nc}\right) $$

1 $$ \mathrm{with}\ {D}^{(i)}\left(\boldsymbol{nc}\right)\ge He(t), $$

where *He*(*t*), ∀ *t* ∈ [0, *t*^∗^] is the minimal genomewide diversity constraint at time *t*. The evolution of diversity along time was controlled by the targeted diversity trajectory, i.e. *He*(*t*), ∀ *t* ∈ [0, *t*^∗^] where *t*^∗^ ∈ *ℕ*^∗^ is the time horizon when the diversity *He*(*t*^∗^) = *He*^∗^ should be reached. For the external and the commercial benchmark without introduction breeding programs, we considered *He*^∗^ = 0.10 and *He*^∗^ = 0.01 reached after 60 years, respectively. As in Allier et al. [5], the constraint on *D*^(*i*)^ followed a linear trajectory over time:

2 $$ He(t)=\left\{\begin{array}{c}{He}^0+\frac{t}{t^{\ast }}\left({He}^{\ast }-{He}^0\right),\forall t\in \left[0,{t}^{\ast}\right]\\ {}{He}^{\ast },\forall t>{t}^{\ast}\end{array}\right), $$

where *He*^0^ is the initial diversity at *t* = 0, i.e. at the end of burn-in.

For the commercial breeding program with introductions, we maintained the genomewide diversity constant after the end of burn-in, i.e. *He*(*t*) = *He*^0^, ∀ *t* ∈ [0, *t*^∗^]. Thus, the UCPC based OCS selected introduction crosses (i.e. DxE if no bridging and DExE if bridging) when necessary to maximize the performance while keeping genomewide diversity constant (Eq. 1). In case of bridging, we completed the 15 selected breeding crosses with 5 bridging crosses (DxE, Fig. 7d) that maximized the following function on the full set of |***nc***| ***=*** 20 crosses:

3 $$ \underset{\boldsymbol{nc}}{\max }\ \alpha\ {V}^{(i)\ast}\left(\boldsymbol{nc}\right)+\left(1-\alpha \right)\ {D}^{(i)\ast}\left(\boldsymbol{nc}\right), $$

where ${V}^{(i)\ast}\left(\boldsymbol{nc}\right)=\frac{V^{(i)}\left(\boldsymbol{nc}\right)-{V}^{(i)}\left({\boldsymbol{nc}}_D^{\ast}\right)}{V^{(i)}\left({\boldsymbol{nc}}_V^{\ast}\right)-{V}^{(i)}\left({\boldsymbol{nc}}_D^{\ast}\right)}$ and ${D}^{(i)\ast}\left(\boldsymbol{nc}\right)=\frac{D^{(i)}\left(\boldsymbol{nc}\right)-{D}^{(i)}\left({\boldsymbol{nc}}_V^{\ast}\right)}{D^{(i)}\left({\boldsymbol{nc}}_D^{\ast}\right)-{D}^{(i)}\left({\boldsymbol{nc}}_V^{\ast}\right)}$ with ${\boldsymbol{nc}}_V^{\ast }$ and ${\boldsymbol{nc}}_D^{\ast }$ the lists of crosses that maximize the performance (*V*) and the diversity (*D*), respectively, considering a within family selection intensity of *i*. *α* ∈ [0, 1] is the relative weight given to performance compared to diversity. A differential evolution (DE) algorithm was used to find Pareto-optimal solutions of Eq. 1 and Eq. 3 [35, 36, 64].

### Advantages of pre-breeding and bridging

We compared different commercial breeding programs at a constant cost (i.e. total of 1600 DH/year) with recurrent introductions (i) either direct or with a bridging step and (ii) considering three types of potential donors, resulting in the six genetic base broadening scenarios: *Bridging_Panel*, *Nobridging_Panel*, *Bridging_20y*, *Nobridging_20y*, *Bridging_5y*, *Nobridging_5y*. We ran 10 independent simulation replicates of the external program that generated donors, the commercial benchmark program without introductions, and the six genetic base broadening scenarios. Note that at a given simulation replicate the commercial breeding program accessed the potential donors released by the corresponding external breeding program simulation replicate.

We followed several indicators in the breeding families (i.e. E progeny, Fig. 7). At each generation *T* ∈ [0, 60] with *T* = 0 corresponding to the last burn-in generation, we computed the mean true breeding value (TBV) of E progeny *μ*(*T*) = *mean*(*TBV*(*T*)) and of the 10 most performing E progeny ${\mu}_{10}(T)= mean\left(\underset{10}{\max}\left( TBV(T)\right)\right)$ as a proxy of the performance that could be achieved at the commercial level by releasing these lines as varieties. We also measured the frequency of the favorable allele in the E progeny *p*_*j*_(*T*) at each QTL *j* among the 1000 QTLs. We further focused on the QTLs where the favorable allele was rare at the end of burn-in, i.e. *p*_*j*_(0) ≤ 0.05. The results were averaged and standard errors were computed over 10 independent replicates.

### Effect of a joint genomic selection model for bridging and breeding

For the three scenarios with bridging, we investigated the advantage of a single TS grouping 3600 DE and 1200 E progeny to predict both breeding and bridging families. These three additional scenarios were referred to as *Bridging_Panel (Single TS)*, *Bridging_20y (Single TS)* and *Bridging_5y (Single TS)*. Every generation, we defined the prediction accuracies as the correlation between true breeding values and GEBVs ($cor\left(u,\hat{u}\right)$) within breeding elite families (ExE), breeding introduction families (DExE) and bridging families (DxE). The prediction accuracies were averaged over the 10 replicates and further averaged over the 60 generations. Note that considering a single GS model at constant cost yielded not only a broader but also a larger training set (4800 DH progeny instead of 3600 DH progeny for elite GS or 1200 DH progeny for bridging GS, Fig. 7).

We further investigated the effect of the proportion of DE and E progeny in the TS at constant size on within ExE and DExE family selection accuracy. We considered the 1200 DE and 3600 E progeny genotypes and phenotypes simulated at generations 18, 19, 20 in the first replicate of scenario *Bridging_20y*. We further selected the 5% DH per family with the highest GEBVs obtained using a GS model trained on all 4800 progeny genotypes and phenotypes. These were randomly crossed to generate 50 elite (ExE) and 50 introduction (DExE) families of 80 DH progeny. These families were considered as the validation set (VS). We randomly sampled among the 4800 DH progeny different TS of variable sizes and compositions (Table 2) and we evaluated the within elite (ExE) and introduction (DExE) family prediction accuracy ($cor\left(u,\hat{u}\right)$). We also evaluated the within family variance prediction accuracy as the correlation between the variance of true breeding values and the estimated variance ($cor\left(\sigma, \hat{\sigma}\right)$). We reported results for 20 independent samples.

**Table 2:** Description of the compared training sets

	TS name	Number of E	Number of DE
**Full TS**	Pure E (3,600)	3600	0
	Pure DE (1200)	0	1200
	1/4 - DE (4800)	3600	1200
**Constant size (1200)**	Pure E (1,200)	1200	0
	1/4 - DE (1200)	900	300
**Constant size (3600)**	1/3 - DE (3600)	2400	1200
	1/4 - DE (3600)	2700	900
	1/6 - DE (3600)	3000	600
	1/12 – DE (3600)	3300	300
	1/24 - DE (3600)	3450	150
	1/36 - DE (3600)	3500	100

The full training sets considering all available progeny of the last three generations and training sets at constant size (1200 progeny or 3600 progeny) with variable proportion of DE progeny

## Supplementary information

**Additional file 1.** contains additional information on the simulation of genotypes, the simulation of phenotypes and the genomewide prediction model considered.

**Additional file 2.** details the usefulness criterion parental contributions based optimal cross selection methodology.

**Additional file 3: Supplementary Tables** contain the supplemental **Table S1.** (Mean progeny performance at different generations); **Table S2.** (Mean performance of the ten best progeny at different generations); **Table S3.** (Frequency of the rare favorable alleles in the bridging population at different generations).

**Additional file 4:Supplementary Figures** contain the supplemental **Figure S1.** (Evolution of the additive genetic variance intra- and inter-family components in the breeding population); **Figure S2.** (Summary statistics on the introduction crosses); **Figure S3.** (Effect of TS composition on intra-family prediction accuracies); **Figure S4.** (Evolution of the breeding population over generations for two different weightings α).

## Data Availability

Data used in this manuscript are publically available at 10.25387/g3.7405892 and the R code of key functions can be found at 10.3389/fgene.2019.01006.

## Abbreviations

CDmean: Mean coefficient of determination
DE: Differential evolution
DH: Doubled haploid
G-BLUP: Genomic best linear unbiased predictor
GEBV: Genomic estimated breeding value
GEM: Germplasm enhancement of maize project
GS: Genomic selection
He: Expected heterozygosity
HOPE: Hierarchical open-ended population enrichment
LAMP: Latin American maize project
OCS: Optimal cross selection
OPVs: Open pollinated varieties
PVPA: Plant variety protection act
QTLs: Quantitative trait loci
SeeD: Seeds of discovery project
SNP: Single nucleotide polymorphism
TBV: True breeding value
TS: Training set
UCPC: Usefulness criterion parental contribution
UPOV: Union for the protection of new varieties
US: United States of America
VS: Validation set
