# Supplementary material for: Optimized breeding strategies to harness genetic resources with different performance levels
Source: BMC Genomics. 2020 May 11;21:349. doi: 10.1186/s12864-020-6756-0 (PMC7216646; doi:10.1186/s12864-020-6756-0)
Supplement: Supplementary file 1 — Additional file 1. contains additional information on the simulation of genotypes, the simulation of phenotypes and the genomewide prediction model considered. [file 12864_2020_6756_MOESM1_ESM.pdf]

Additional File 1

Simulation of progeny genotypes and phenotypes

Doubled haploid (DH) progeny genotypes were simulated considering meiosis events without crossover interference. The number of chiasmata was drawn from a Poisson distribution with λ equal to the chromosome length in Morgan, and crossover positions were determined using the recombination frequency obtained using the Haldane mapping function (Haldane 1919).

For phenotyping, we considered environmental effects sampled from a normal distribution of mean zero and variance 25 and did not consider genotype by environment interactions. Each generation was evaluated in $N_{loc} = 4$ locations in one year, i.e. four environments. Environmental errors were sampled from a normal distribution with mean zero and an error variance σ_ϵ^2 defined by the initial repeatability in the founder population $r = \frac{\sigma_G^2}{\sigma_G^2+\sigma_\epsilon^2} = 0.40$. This led to a heritability in the founder population of $h^2 = \frac{\sigma_G^2}{\sigma_G^2+\sigma_\epsilon^2/N_{loc}} = 0.73$ and $h^2 = 0.42$ at the end of burn-in in commercial breeding scenarios.

Genomewide prediction model

The genomic estimated breeding values of progeny (GEBV, $\hat{u}$) were estimated in Model 1 S1 fitted using mixed model software blup-f 90 (Misztal 2008) with AI-REML variance component estimates:

$$\boldsymbol{Y} = \boldsymbol{1}\mu + \boldsymbol{E\beta_{Env}} + \boldsymbol{Wu} + \boldsymbol{\epsilon}, \text{(Model 1 S1)}$$

where $\boldsymbol{Y}$ is the vector of phenotypic values, μ is the intercept, $\boldsymbol{E}$ is the incidence matrix for environmental effects, $\boldsymbol{\beta_{Env}}$ is the vector of environmental fixed effects, $\boldsymbol{W}$ is the incidence matrix of individual breeding value random effects $\boldsymbol{u}$, $\boldsymbol{u} \sim N(\boldsymbol{0}, \sigma_G^2\boldsymbol{G})$ is the vector of breeding value random effects with $\boldsymbol{G}$ the genomic relationship matrix and $\boldsymbol{\epsilon}$ is the vector of independent residual random terms $\boldsymbol{\epsilon} \sim N(\boldsymbol{0}, \sigma_\epsilon^2\boldsymbol{I})$. $\boldsymbol{G}$ was estimated using the 2,000 non causal loci:

$$\boldsymbol{G} = \frac{\boldsymbol{ZZ'}}{tr(\boldsymbol{ZZ'})/n}$$

where $\boldsymbol{Z}$ contains the centered allele counts, with elements computed as $x_{ij} + 1 - 2p_j$, where the element $x_{ij} \in \{-1,1\}$ is the genotype for individual i at non causal locus j and p_j is the frequency of the allele for which the homozygous genotype is coded 1 at non causal locus j. $tr(\boldsymbol{ZZ'})$ is the trace of $\boldsymbol{ZZ'}$ and $tr(\boldsymbol{ZZ'})/n$ forces the diagonal of $\boldsymbol{G}$ to be 1 on average (Legarra *et al.* 2009; Forni *et al.* 2011). Estimated marker effects $\widehat{\boldsymbol{\beta}}$ were obtained by back-solving: $\widehat{\boldsymbol{\beta}} = \boldsymbol{Z}'(\boldsymbol{ZZ}')^{-1}\widehat{\boldsymbol{u}}$ (Wang *et al.* 2012). The prediction accuracy was defined as $cor(\boldsymbol{u}, \widehat{\boldsymbol{u}})$ with $\boldsymbol{u}$ and $\widehat{\boldsymbol{u}}$ the vectors of true breeding values and genomic estimated breeding values, respectively.

Literature cited

Forni S., I. Aguilar, and I. Misztal, 2011 Different genomic relationship matrices for single-step analysis using phenotypic, pedigree and genomic information. Genet. Sel. Evol. 43: 1.

Haldane J., 1919 The combination of linkage values, and the calculation of distances between the loci of linked factors. J Genet 8: 299–309.

Legarra A., I. Aguilar, and I. Misztal, 2009 A relationship matrix including full pedigree and genomic information. J. Dairy Sci. 92: 4656–4663.

Misztal I., 2008 Reliable computing in estimation of variance components. J. Anim. Breed. Genet. 125: 363–370.

Wang H., I. Misztal, I. Aguilar, A. Legarra, and W. M. Muir, 2012 Genome-wide association mapping including phenotypes from relatives without genotypes. Genet. Res. 94: 73–83.
