# Supplementary material for: Optimized breeding strategies to harness genetic resources with different performance levels
Source: BMC Genomics. 2020 May 11;21:349. doi: 10.1186/s12864-020-6756-0 (PMC7216646; doi:10.1186/s12864-020-6756-0)
Supplement: Supplementary file 2 — Additional file 2. details the usefulness criterion parental contributions based optimal cross selection methodology. [file 12864_2020_6756_MOESM2_ESM.pdf]

Additional File 2

We applied the Usefulness Criterion Parental Contributions approach (UCPC) proposed by Allier *et al.* (2019a) and further extended in Allier *et al.* (2019b) to evaluate the interest of a set of two-way crosses regarding the performance and the diversity in the best fraction of the progeny of each cross.

Prediction of the mean expected breeding value and parental contributions in the selected fraction of progeny

Considering two inbred lines P_1 and P_2 and the cross P_1 x P_2 and $(\boldsymbol{x}_1, \boldsymbol{x}_2)'$ denotes their (2 x m)-dimensional genotyping matrix at the $m = 2{,}000$ SNP markers. $\boldsymbol{x_p}$ denotes the (m x 1)-dimensional genotype vector of parent $P_{p\in\{1,2\}}$ with the jth element coded as 1 or −1 for the genotypes AA or aa at QTL j. Following Lehermeier *et al.* (2017), the DH progeny mean and progeny variance of the breeding values in the progeny before selection can be computed as:

$$\hat{\mu}_T = 0.5\ (\boldsymbol{x'}_1\widehat{\boldsymbol{\beta}} + \boldsymbol{x'}_2\widehat{\boldsymbol{\beta}}), \text{ (Eq. 1a)}$$

$$\hat{\sigma}_T^2 = \widehat{\boldsymbol{\beta}}'\ \boldsymbol{\Sigma}\ \widehat{\boldsymbol{\beta}}, \text{ (Eq. 1b)}$$

where $\widehat{\boldsymbol{\beta}}$ is (m x 1)-dimensional vector of estimated marker effects and $\boldsymbol{\Sigma}$ is the (m x m)-dimensional variance covariance matrix of marker genotypes in DH progeny defined in Lehermeier *et al.* (2017). We define the (m x 1)-dimensional vector $\boldsymbol{\beta}_{C1}$ to follow P_1 genome contribution to progeny as $\boldsymbol{\beta}_{C1} = \frac{\boldsymbol{x}_1 - \boldsymbol{x}_2}{(\boldsymbol{x}_1 - \boldsymbol{x}_2)'(\boldsymbol{x}_1 - \boldsymbol{x}_2)}$. The mean and variance of P_1 contribution in the progeny before selection are computed as:

$$\mu_{C1} = 0.5\ (\boldsymbol{x'}_1\boldsymbol{\beta}_{C1} + \boldsymbol{x'}_2\boldsymbol{\beta}_{C1} + 1), \text{ (Eq. 2a)}$$

$$\sigma_{C1}^2 = \boldsymbol{\beta}'_{C1}\ \boldsymbol{\Sigma}\ \boldsymbol{\beta}_{C1}. \text{ (Eq. 2b)}$$

The progeny mean for P_2 contribution is then $\mu_{C2} = 1 - \mu_{C1}$.

Following Allier *et al.* (2019a), the covariance between the breeding values and P_1 contribution in progeny is:

$$\hat{\sigma}_{T,C1} = \widehat{\boldsymbol{\beta}}'\ \boldsymbol{\Sigma}\ \boldsymbol{\beta}_{C1}. \text{ (Eq. 3)}$$

The expected mean breeding value of the selected fraction of progeny, i.e. usefulness criterion (Schnell and Utz 1975), of the cross P_1 x P_2 is:

$$\widehat{UC}^{(i,h)} = \hat{\mu}_T + ih\hat{\sigma}_T, \text{ (Eq. 4)}$$

where i is the within family selection intensity and h the within family selection accuracy. The correlated responses to selection on P_1 and P_2 contributions to the selected fraction of progeny are (Falconer and Mackay 1996):

$$\hat{c}_1^{(i,h)} = \mu_{C1} + ih\frac{\hat{\sigma}_{T,C1}}{\hat{\sigma}_T} \text{ and } \hat{c}_2^{(i,h)} = 1 - \hat{c}_1^{(i,h)}. \text{ (Eq. 5)}$$

Optimal cross selection accounting for within family variance

Considering N homozygote candidate parents, $N(N-1)/2$ two-way crosses are possible. We define a crossing plan $\boldsymbol{nc}$ as a set of $|nc|$ crosses out of possible two-way crosses, giving the index of selected crosses, i.e. with the ith element $nc\,(i) \in [1, N(N-1)/2]$. The $(N \text{ x } 1)$-dimensional vector of candidate parents estimated contributions in the selected fraction of progeny of each cross $\hat{\boldsymbol{c}}^{(i,h)}$ is:

$$\hat{\boldsymbol{c}}^{(i,h)} = \frac{1}{|nc|}\left(\boldsymbol{Z_1}\hat{\boldsymbol{c}}_{\boldsymbol{1}}^{(i,h)} + \boldsymbol{Z_2}\hat{\boldsymbol{c}}_{\boldsymbol{2}}^{(i,h)}\right), \text{ (Eq. 6)}$$

where $\boldsymbol{Z_1}$ (respectively $\boldsymbol{Z_2}$) is a $(N \text{ x } |nc|)$-dimensional design matrix that links each N candidate parent to the first (respectively second) parent in the set of crosses $\boldsymbol{nc}$, $\hat{\boldsymbol{c}}_{\boldsymbol{1}}^{(i,h)}$ (respectively $\hat{\boldsymbol{c}}_{\boldsymbol{2}}^{(i,h)}$) is a $(|nc| \text{ x } 1)$-dimensional vector containing the estimated contributions of the first (respectively second) parent to the selected fraction of the progeny of the crosses in $\boldsymbol{nc}$.

The expected performance $V(\boldsymbol{nc})$ for this set of two-way crosses is defined as the expected mean performance of the selected DH progeny, i.e. usefulness criterion:

$$\hat{V}^{(i,h)}(\boldsymbol{nc}) = \frac{1}{|\boldsymbol{nc}|}\sum_{j\in\boldsymbol{nc}} \widehat{\boldsymbol{UC}}^{(i,h)}(j). \text{ (Eq. 7)}$$

The constraint on diversity $\widehat{D}^{(i,h)}(\boldsymbol{nc})$ in the selected progeny is:

$$\widehat{D}^{(i,h)}(\boldsymbol{nc}) = 1 - \hat{\boldsymbol{c}}^{(i,h)'}\boldsymbol{K}\,\hat{\boldsymbol{c}}^{(i,h)}, \text{ (Eq. 8)}$$

where $\boldsymbol{K}$ is the $(N \text{ x } N)$-dimensional identity by state (IBS) coancestry matrix at markers between the N candidates. Allier *et al.* (2019b) showed that $\widehat{D}^{(i,h)}(\boldsymbol{nc})$ is a good proxy of the genomewide diversity

in the selected fraction of progeny $He^{(i,h)} = \frac{1}{m}\sum_{j=1}^{m} 2p_j^{(i,h)}(1 - p_j^{(i,h)})$ where $p_j^{(i,h)}$ is the frequency of the genotypes AA at marker j in the selected fraction of progeny.

Literature cited

Allier A., L. Moreau, A. Charcosset, S. Teyssèdre, and C. Lehermeier, 2019a Usefulness Criterion and Post-selection Parental Contributions in Multi-parental Crosses: Application to Polygenic Trait Introgression. G3 Genes Genomes Genet. 9: 1469–1479.

Allier A., C. Lehermeier, A. Charcosset, L. Moreau, and S. Teyssèdre, 2019b Improving Short- and Long-Term Genetic Gain by Accounting for Within-Family Variance in Optimal Cross-Selection. Front. Genet. 10.

Falconer D. S., and T. F. C. Mackay, 1996 *Introduction to Quantitative Genetics. 4th ed.* Pearson, Harlow, England.

Lehermeier C., S. Teyssèdre, and C.-C. Schön, 2017 Genetic Gain Increases by Applying the Usefulness Criterion with Improved Variance Prediction in Selection of Crosses. Genetics 207: 1651–1661.

Schnell F., and H. Utz, 1975 F1-Leistung und Elternwahl in der Züchtung von Selbstbefruchtern., pp. 243–248 in *Bericht über die Arbeitstagung der Vereinigung österreichischer Pflanzenzüchter.*, BAL Gumpenstein, Austria.
