# Supplementary material for: Optimized breeding strategies to harness genetic resources with different performance levels
Source: BMC Genomics. 2020 May 11;21:349. doi: 10.1186/s12864-020-6756-0 (PMC7216646; doi:10.1186/s12864-020-6756-0)
Supplement: Supplementary file 3 — Additional file 3: Supplementary Tables contain the supplemental Table S1. (Mean progeny performance at different generations); Table S2. (Mean performance of the ten best progeny at different generations); Table S3. (Frequency of the rare favorable alleles in the bridging population at different generations). [file 12864_2020_6756_MOESM3_ESM.docx]

**Supplementary Tables**

**Table S1** Mean breeding population performance ($\mu$) at different generations (5, 10, 20, 30, 40, 50 and 60 years). In brackets is given the standard error (standard deviation divided by the square root of the number of independent replicates: $\sqrt{10}$).

| **Scenario** | **Population mean performance (**$\boldsymbol{\mu}$**)** | | | | | | |
| --- | --- | --- | --- | --- | --- | --- | --- |
|  | **5 years** | **10 years** | **20 years** | **30 years** | **40 years** | **50 years** | **60 years** |
| **Benchmark** | 9.239  (+/- 0.237) | 14.913 (+/- 0.645) | 23.182 (+/- 1.446) | 30.006 (+/- 1.319) | 34.741 (+/- 1.329) | 37.486 (+/- 1.487) | 38.837 (+/- 1.563) |
| **Nobridging_Panel** | 8.168  (+/- 0.282) | 13.022 (+/- 0.890) | 12.589 (+/- 0.988) | 9.355 (+/- 3.147) | 11.059 (+/- 0.933) | 10.581 (+/- 0.797) | 9.651  (+/- 0.958) |
| **Bridging_Panel** | 8.688  (+/- 0.329) | 13.653 (+/- 0.867) | 25.212 (+/- 1.314) | 35.770 (+/- 1.077) | 42.828 (+/- 1.091) | 47.681 (+/- 1.256) | 52.110 (+/- 0.886) |
| **Bridging_Panel (Single TS)** | 9.635  (+/- 0.267) | 13.988 (+/- 1.599) | 29.292 (+/- 0.802) | 38.398 (+/- 1.133) | 45.374 (+/- 1.090) | 52.036 (+/- 1.262) | 57.067 (+/- 1.444) |
| **Nobridging_20y** | 8.383  (+/- 0.271) | 12.083 (+/- 2.563) | 16.818 (+/- 2.397) | 33.546 (+/- 1.519) | 47.346 (+/- 1.096) | 58.628 (+/- 1.087) | 66.944 (+/- 0.849) |
| **Bridging_20y** | 8.687  (+/- 0.293) | 13.045 (+/- 1.328) | 27.987 (+/- 0.840) | 40.296 (+/- 1.010) | 51.468 (+/- 0.957) | 60.939 (+/- 1.010) | 69.154 (+/- 0.868) |
| **Bridging_20y (Single TS)** | 9.430  (+/- 0.229) | 14.431 (+/- 1.419) | 30.497 (+/- 1.135) | 43.238 (+/- 1.384) | 54.373 (+/- 1.220) | 64.066 (+/- 1.121) | 71.130 (+/- 1.028) |
| **Nobridging_5y** | 9.820  (+/- 0.358) | 16.356 (+/- 0.954) | 34.541 (+/- 0.980) | 49.837 (+/- 0.911) | 60.424 (+/- 0.952) | 68.524 (+/- 0.918) | 74.662 (+/- 0.938) |
| **Bridging_5y** | 10.152  (+/- 0.368) | 18.146 (+/- 1.180) | 34.900 (+/- 0.905) | 49.131 (+/- 1.164) | 60.542 (+/- 1.021) | 68.184 (+/- 0.916) | 74.074 (+/- 0.869) |
| **Bridging_5y (Single TS)** | 12.348  (+/- 0.622) | 19.246 (+/- 1.868) | 40.111 (+/- 1.149) | 53.981 (+/- 1.047) | 63.540 (+/- 1.052) | 70.603 (+/- 1.160) | 75.749 (+/- 1.093) |

**Table S2** Mean performance of the ten best progeny ($\mu_{10}$) at different generations (5, 10, 20, 30, 40, 50 and 60 years). In brackets is given the standard error (standard deviation divided by the square root of the number of independent replicates: $\sqrt{10}$).

| **Scenario** | **Ten best mean performance (**$\boldsymbol{\mu}_{\boldsymbol{10}}$**)** | | | | | | |
| --- | --- | --- | --- | --- | --- | --- | --- |
|  | **5 years** | **10 years** | **20 years** | **30 years** | **40 years** | **50 years** | **60 years** |
| **Benchmark** | 15.746  (+/- 0.391) | 20.544 (+/- 0.945) | 27.346 (+/- 1.527) | 33.169 (+/- 1.360) | 37.020 (+/- 1.365) | 38.761 (+/- 1.505) | 39.567 (+/- 1.571) |
| **Nobridging_Panel** | 15.802  (+/- 0.341) | 20.776 (+/- 0.499) | 27.215 (+/- 0.740) | 25.015 (+/- 2.638) | 28.625 (+/- 0.775) | 29.255 (+/- 0.855) | 29.767 (+/- 1.108) |
| **Bridging_Panel** | 15.605  (+/- 0.477) | 21.148 (+/- 0.773) | 33.215 (+/- 1.257) | 43.408 (+/- 1.379) | 51.348 (+/- 1.361) | 57.376 (+/- 1.447) | 61.763 (+/- 1.298) |
| **Bridging_Panel (Single TS)** | 16.727  (+/- 0.527) | 21.063 (+/- 1.530) | 35.619 (+/- 0.843) | 44.730 (+/- 1.399) | 52.026 (+/- 1.352) | 57.796 (+/- 1.524) | 63.699 (+/- 1.698) |
| **Nobridging_20y** | 15.991  (+/- 0.476) | 19.827 (+/- 1.855) | 25.694 (+/- 1.882) | 40.211 (+/- 1.487) | 53.449 (+/- 1.087) | 64.336 (+/- 1.138) | 72.258 (+/- 0.978) |
| **Bridging_20y** | 15.793  (+/- 0.357) | 20.123 (+/- 1.240) | 34.896 (+/- 1.009) | 46.471 (+/- 1.104) | 57.205 (+/- 1.062) | 66.774 (+/- 1.115) | 74.413 (+/- 0.932) |
| **Bridging_20y (Single TS)** | 16.364  (+/- 0.520) | 21.588 (+/- 1.342) | 36.989 (+/- 1.179) | 49.263 (+/- 1.460) | 59.814 (+/- 1.252) | 69.221 (+/- 1.233) | 75.747 (+/- 1.052) |
| **Nobridging_5y** | 17.077  (+/- 0.476) | 23.499 (+/- 0.730) | 41.389 (+/- 0.978) | 55.828 (+/- 0.910) | 65.767 (+/- 1.036) | 73.923 (+/- 1.005) | 79.776 (+/- 1.058) |
| **Bridging_5y** | 17.745  (+/- 0.577) | 25.232 (+/- 1.262) | 41.448 (+/- 1.004) | 55.119 (+/- 1.246) | 66.063 (+/- 0.975) | 73.297 (+/- 0.803) | 78.694 (+/- 0.885) |
| **Bridging_5y (Single TS)** | 20.110  (+/- 0.725) | 25.900 (+/- 2.208) | 46.272 (+/- 1.124) | 59.490 (+/- 1.074) | 68.559 (+/- 1.080) | 75.284 (+/- 1.289) | 80.058 (+/- 1.125) |

**Table S3** Frequency of the rare favorable allele in the bridging population at different generations (5, 10, 20, 40, 50 and 60 years). The rare favorable alleles were defined with a frequency $\leq0.05$ at the end of burn-in and concerned on average 269.9 (+/- 23.6) QTLs out of the 1,000 QTLs. In brackets is given the standard error (standard deviation divided by the square root of the number of independent replicates:$\sqrt{10}$).

| **Scenario** | **Mean frequency of rare favorable allele** | | | | | | |
| --- | --- | --- | --- | --- | --- | --- | --- |
|  | **5 years** | **10 years** | **20 years** | **30 years** | **40 years** | **50 years** | **60 years** |
| **Benchmark** | 0.005 (+/- 0.001) | 0.006 (+/- 0.002) | 0.010 (+/- 0.004) | 0.013 (+/- 0.005) | 0.015 (+/- 0.006) | 0.016 (+/- 0.006) | 0.016 (+/- 0.006) |
| **Nobridging_Panel** | 0.019 (+/- 0.002) | 0.036 (+/- 0.004) | 0.053 (+/- 0.006) | 0.069 (+/- 0.013) | 0.063 (+/- 0.007) | 0.065 (+/- 0.007) | 0.068 (+/- 0.007) |
| **Bridging_Panel** | 0.021 (+/- 0.003) | 0.054 (+/- 0.010) | 0.112 (+/- 0.010) | 0.169 (+/- 0.011) | 0.209 (+/- 0.010) | 0.240 (+/- 0.009) | 0.263 (+/- 0.008) |
| **Bridging_Panel (Single TS)** | 0.030 (+/- 0.004) | 0.055 (+/- 0.006) | 0.126 (+/- 0.009) | 0.179 (+/- 0.010) | 0.213 (+/- 0.011) | 0.245 (+/- 0.012) | 0.272 (+/- 0.014) |
| **Nobridging_20y** | 0.017 (+/- 0.002) | 0.047 (+/- 0.010) | 0.088 (+/- 0.014) | 0.160 (+/- 0.010) | 0.245 (+/- 0.012) | 0.313 (+/- 0.012) | 0.358 (+/- 0.010) |
| **Bridging_20y** | 0.022 (+/- 0.002) | 0.053 (+/- 0.007) | 0.116 (+/- 0.011) | 0.186 (+/- 0.014) | 0.253 (+/- 0.009) | 0.309 (+/- 0.009) | 0.361 (+/- 0.009) |
| **Bridging_20y (Single TS)** | 0.029 (+/- 0.004) | 0.068 (+/- 0.006) | 0.145 (+/- 0.013) | 0.215 (+/- 0.013) | 0.282 (+/- 0.012) | 0.342 (+/- 0.011) | 0.380 (+/- 0.010) |
| **Nobridging_5y** | 0.038 (+/- 0.009) | 0.080 (+/- 0.019) | 0.187 (+/- 0.015) | 0.279 (+/- 0.017) | 0.336 (+/- 0.015) | 0.379 (+/- 0.013) | 0.407 (+/- 0.012) |
| **Bridging_5y** | 0.040 (+/- 0.005) | 0.080 (+/- 0.011) | 0.199 (+/- 0.014) | 0.282 (+/- 0.013) | 0.347 (+/- 0.013) | 0.387 (+/- 0.012) | 0.414 (+/- 0.012) |
| **Bridging_5y (Single TS)** | 0.087 (+/- 0.011) | 0.133 (+/- 0.018) | 0.246 (+/- 0.012) | 0.319 (+/- 0.010) | 0.372 (+/- 0.010) | 0.406 (+/- 0.009) | 0.431 (+/- 0.009) |
