# Supplementary material for: Optimized breeding strategies to harness genetic resources with different performance levels
Source: BMC Genomics. 2020 May 11;21:349. doi: 10.1186/s12864-020-6756-0 (PMC7216646; doi:10.1186/s12864-020-6756-0)
Supplement: Supplementary file 4 — Additional file 4:Supplementary Figures contain the supplemental Figure S1. (Evolution of the additive genetic variance intra- and inter-family components in the breeding population); Figure S2. (Summary statistics on the introduction crosses); Figure S3. (Effect of TS composition on intra-family prediction accuracies); Figure S4. (Evolution of the breeding population over generations for two different weightings α). [file 12864_2020_6756_MOESM4_ESM.docx]

**Supplementary Figures**


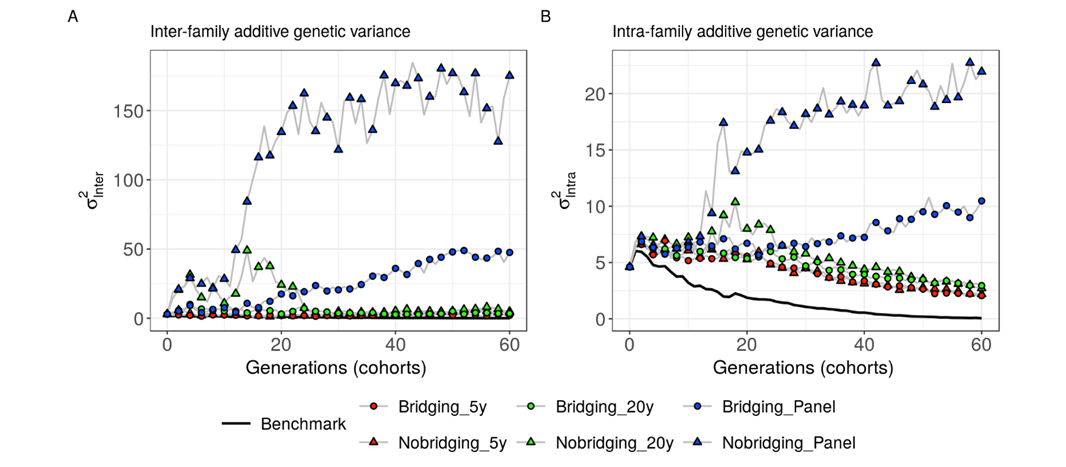
**Figure S1** Evolution of the additive genetic variance intra- and inter-family components in the breeding population. Scenarios considering presence or absence of bridging before introduction and different type of donors (panel, twenty-year old and five-year old donors). (A) Inter-family additive variance and (B) intra-family additive variance.

**
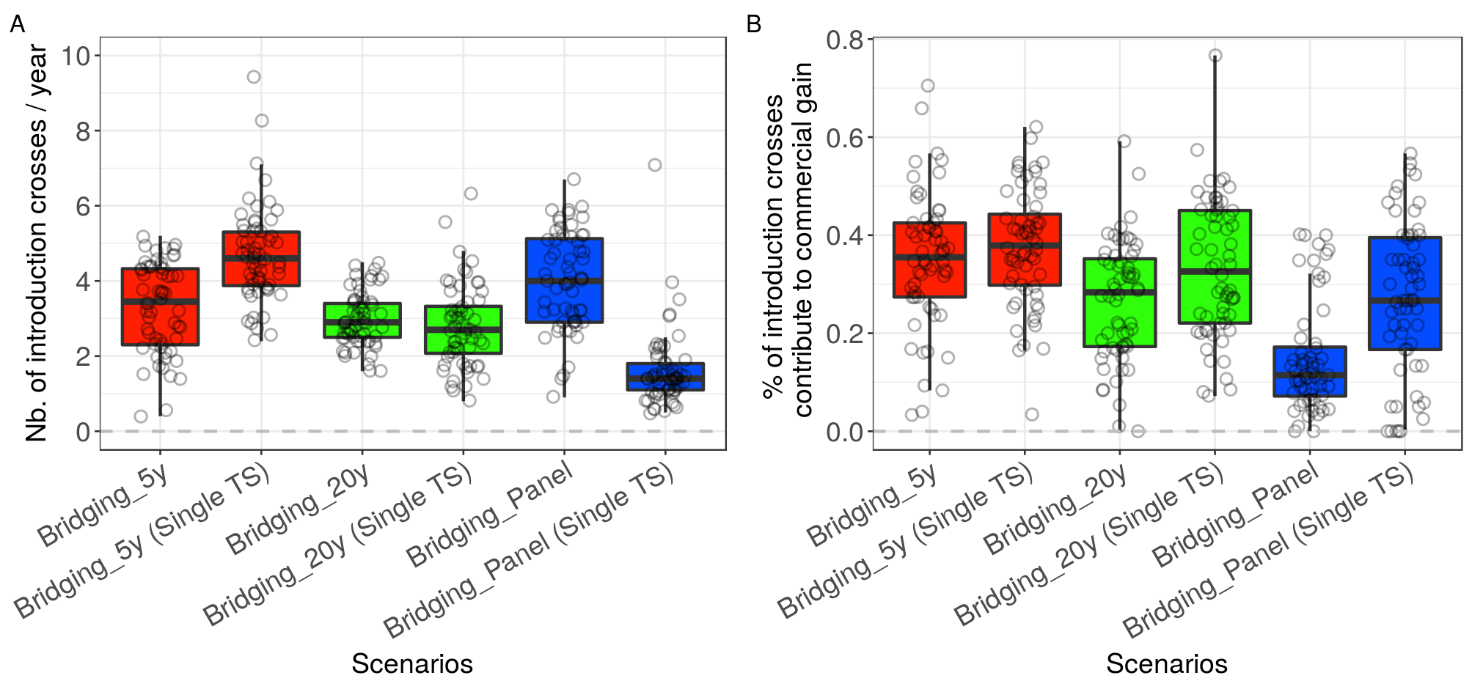
Figure S2** Summary statistics on the introduction crosses. Scenarios considering bridging, different donors (panel, twenty-year old and five-year old donors) and either a single training set (*Single TS*) or two distinct training sets for bridging and breeding (*default*). (A) Number of introduction crosses (DExE) per year and (B) the fraction of the introduction crosses (DExE) that contributed at least in one of the ten best progeny released by the internal breeding program. The distribution over the sixty generations is given after averaging over the ten replicates.

**
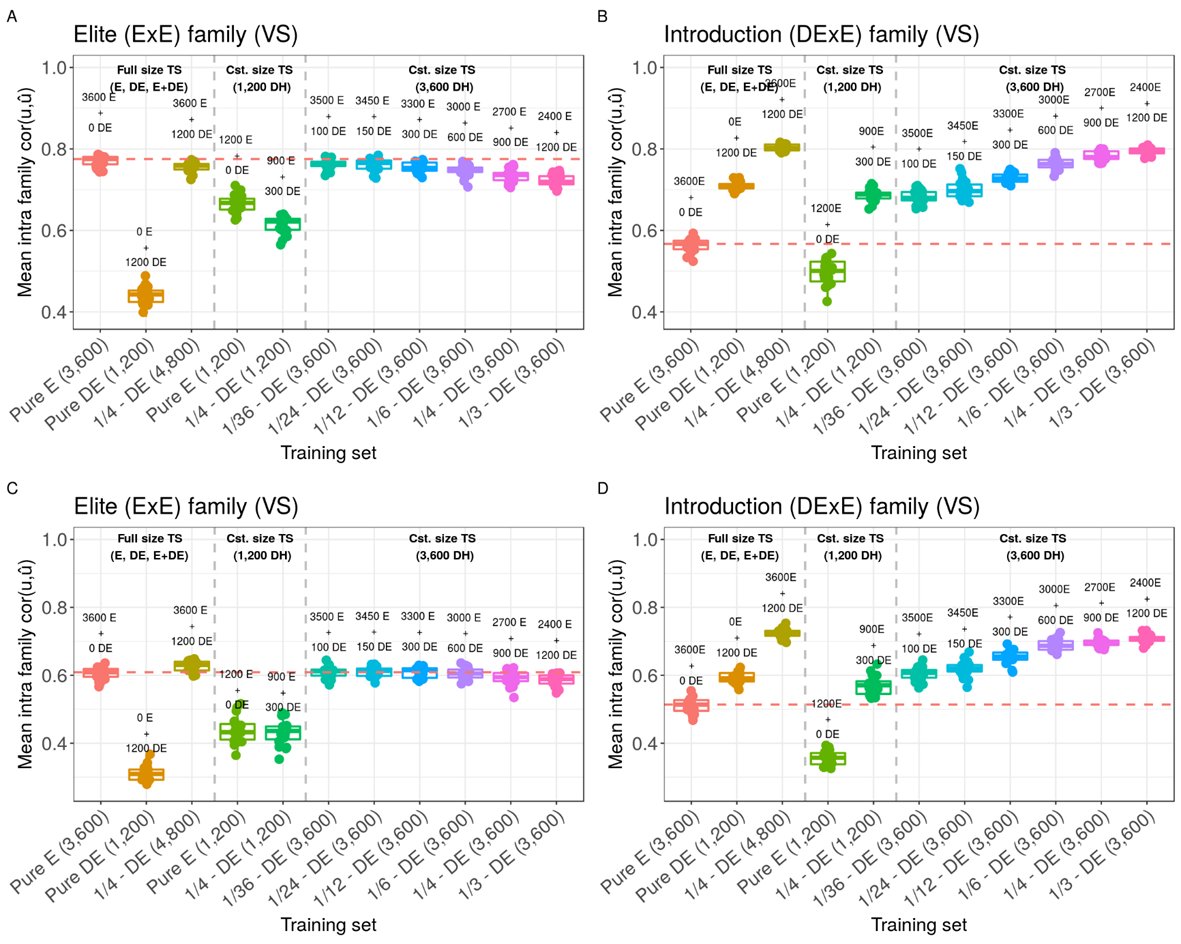
**

**Figure S3** Effect of TS composition on intra-family prediction accuracies ($cor(u,\hat{u})$) considering genotypes simulated at generations 8, 9, 10 (A, B) or 38, 39, 40 (C, D) in the scenario “Bridging_20y”. (A, C) Mean prediction accuracy within 50 elite (ExE) families and (B, D) mean prediction accuracy within 50 introduction (DExE) families. Boxplots represent the results for 20 independent replicates. One can distinguish three training set types (left to right): Full training set considering all 3,600 E progeny (Pure E), all 1,200 DE progeny (Pure DE) and all 3,600 E + 1,200 DE progeny; Training sets at constant size of 1,200 DH for comparison with Pure DE; Training sets at constant size of 3,600 DH and variable proportion of DE progeny for comparison with Pure E. The red dotted line represents the median value for Pure E TS.

**
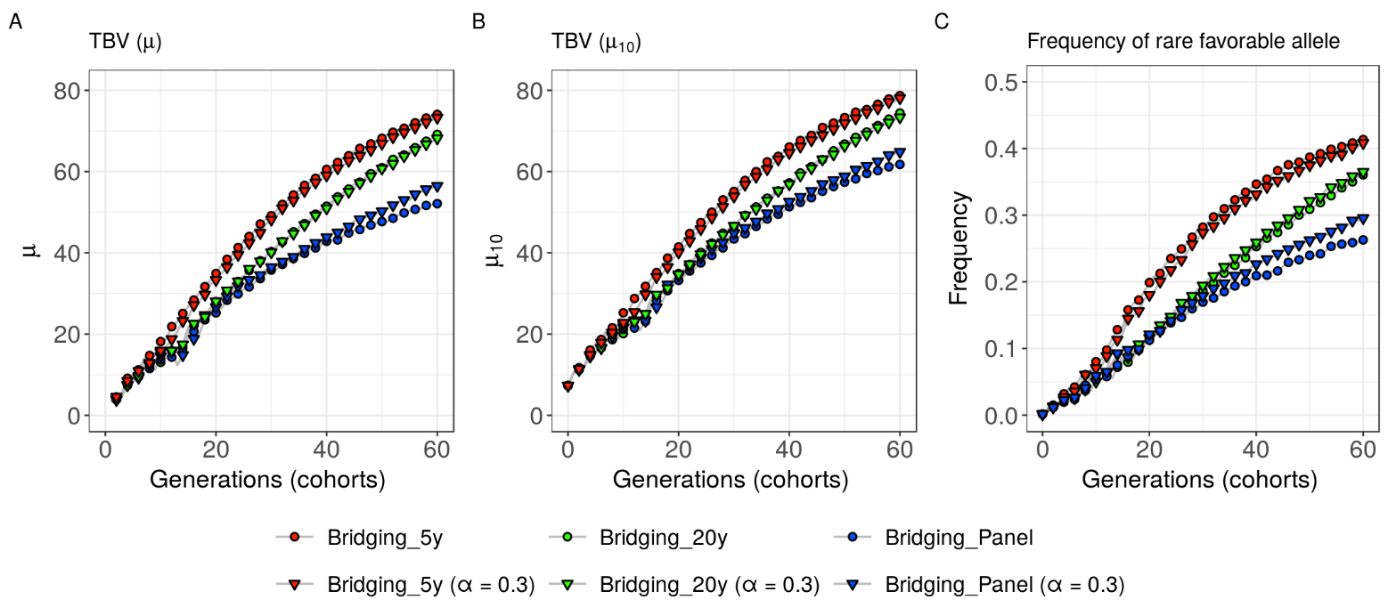
**

**Figure S4** Evolution of the breeding population over generations. Scenarios considering presence of bridging with different type of donors (panel, twenty-year old and five-year old donors) and two weightings for the optimal cross selection in bridging (default is $\alpha=0.7$). (A) Mean breeding population performance ($\mu$), (B) mean performance of the ten best progeny ($\mu_{10}$) and (C) frequency of the favorable alleles that were rare at the end of burn-in (i.e. $p(0)\leq0.05$ corresponding on average to 269.9 +/- 23.6 QTLs).
